# Current Status of the Application of Antimicrobial Peptides and Their Conjugated Derivatives

**DOI:** 10.3390/molecules30153070

**Published:** 2025-07-22

**Authors:** Marcel·lí del Olmo, Cecilia Andreu

**Affiliations:** 1 Departament de Bioquímica i Biologia Molecular, Universitat de València (UVEG), Doctor Moliner 50, 46100 Burjassot, València, Spain; m.del.olmo@uv.es; 2 Department de Química Orgànica, Universitat de València (UVEG), Vicent Andrés Estellés, sn, 46100 Burjassot, València, Spain

**Keywords:** antibiotics, antimicrobial resistance, antimicrobial peptides, antimicrobial peptide conjugates, antimicrobial peptide nanostructures

## Abstract

A significant issue in healthcare is the growing prevalence of antibiotic-resistant strains. Therefore, it is necessary to develop strategies for discovering new antibacterial compounds, either by identifying natural products or by designing semisynthetic or synthetic compounds with this property. In this context, a great deal of research has recently been carried out on antimicrobial peptides (AMPs), which are natural, amphipathic, low-molecular-weight molecules that act by altering the cell surface and/or interfering with cellular activities essential for life. Progress is also being made in developing strategies to enhance the activity of these compounds through their association with other molecules. In addition to identifying AMPs, it is essential to ensure that they maintain their integrity after passing through the digestive tract and exhibit adequate activity against their targets. Significant advances are being made in relation to analyzing various types of conjugates and carrier systems, such as nanoparticles, vesicles, hydrogels, and carbon nanotubes, among others. In this work, we review the current knowledge of different types of AMPs, their mechanisms of action, and strategies to improve performance.

## 1. Introduction

Antimicrobial resistance is a major global health problem that has worsened over the past century. It compromises our ability to treat infectious diseases adequately. The first cases of antimicrobial resistance emerged in the 1940s in response to penicillin and streptomycin [1]. These resistances developed rapidly following the clinical use of these antibiotics during the “Golden Age of Antibiotics”. This prompted the identification and development of new classes of compounds to replace the antibiotics that triggered resistance. However, there has been a lack of new antibiotic development in recent decades [2]. Infections caused by bacterial strains such as MRSA (Methicillin-resistant *Staphylococcus aureus*), CRE (Carbapenem-resistant *Enterobacteriaceae*), and VRE (Vancomycin-resistant *Enterococci*) are of particular global concern [3]. Additionally, multidrug-resistant bacteria, known as “superbugs”, have emerged and are difficult to treat [4]. In 2017, the World Health Organization (WHO) officially recognized antimicrobial resistance as a global health problem, with a particular focus on Gram-negative pathogens and ESKAPE group bacteria (*Enterococcus faecium*, *S. aureus*, *Klebsiella pneumoniae*, *Acinetobacter baumannii*, *Pseudomonas aeruginosa*, and *Enterobacter* spp.). The WHO prioritized developing new drugs against these bacteria. In 2021, the WHO published its annual report on the clinical development of antibiotics, revealing that regulatory agencies have approved very few in recent years and that none of those in development adequately address the problem of resistance [5]. If no progress is made in solving this issue, it is estimated that it could be responsible for 10 million deaths annually by 2050 [3].

**Figure 1:**
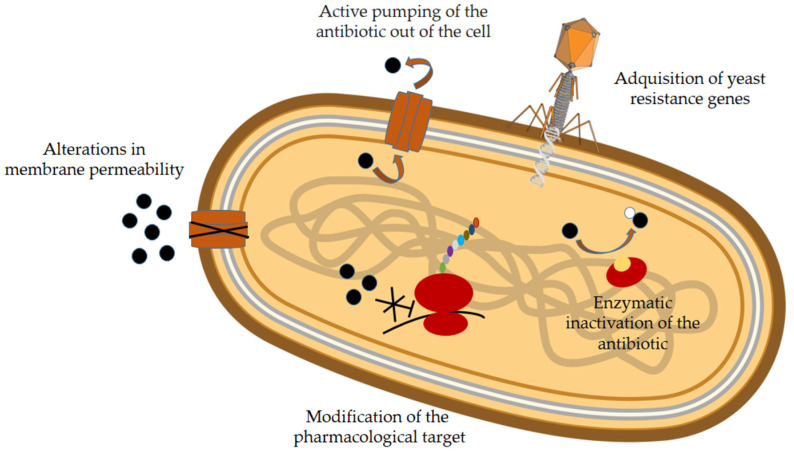
Main mechanisms of antibiotic resistance. Microorganisms can develop several strategies to become resistant to antibiotics. These strategies include the following: (1) Modifying pharmacological targets, such as peptidoglycan or enzymes (e.g., penicillin-binding proteins, DNA gyrase, and DNA topoisomerase). (2) Alterations in membrane permeability due to changes in porin levels, functionality, or selectivity. (3) Active extraction of antibiotics from cells by pumps such as MSF (major facilitator superfamily) and MATE (multidrug and toxic compound extrusion family). (4) Inactivation of enzymes involved in hydrolysis, functional group transfer, or redox processes. (5) Biofilm formation, in which a protective matrix, primarily composed of polysaccharides and proteins, prevents the host from developing immunological mechanisms. (6) The acquisition of resistance genes in the form of free DNA, transposons, plasmids, or integrons through transformation, transduction, or conjugation.

The main causes of antibiotic resistance are the excessive and inappropriate use of antibiotics in humans and livestock, and the lack of precautions in hospitals, which results in healthcare-associated infections [3]. There are two types of bacterial resistance mechanisms: intrinsic and extrinsic. Intrinsic mechanisms are determined by the characteristics of each bacterium, while extrinsic mechanisms result from changes in the genome of these microorganisms due to mutations or the acquisition of exogenous DNA [6]. A thorough description of these processes can be found in several revisions, including those of Martinez (2014) [7] and Gillings et al. (2017) [8]. These mechanisms include modification of the pharmacological target, alterations in membrane permeability, active pumping of the antibiotic out of the cell, enzymatic inactivation, biofilm formation, and acquisition of resistance genes (Figure 1 and Table 1).

Although the generation of antimicrobial resistance seems inevitable, different strategies are being developed to combat it [3,9]. These approaches include discovering new antibiotics, developing alternative therapies such as gene therapy, improving diagnostics to rapidly identify specific pathogens and their antibiotic susceptibility, and implementing programs to educate clinicians and the general public about controlling antibiotic use.

The urgent need to find new compounds with antimicrobial activity requires an intensive search for new molecules that can be used in therapy. These compounds must exhibit broad antimicrobial activity and possess characteristics that distinguish them from existing compounds. They must also be less prone to resistance development. These principles form the basis of alternative therapies under investigation, including those involving the antimicrobial properties of metallic nanoparticles [10,11,12], phytochemicals (such as phenolic acids, flavonoids, tannins, and stilbene) [13,14], bacteriophages [15,16] and monoclonal antibodies [17,18]. Additionally, preventive antibacterial vaccines are being developed. Some of these vaccines are currently in various phases of clinical development [19]. In addition to these strategies, there has been significant interest in compounds known as antimicrobial peptides (AMPs). This work reviews the use of AMPs as an alternative to conventional antibiotics, which have become ineffective due to the progress of resistance. It also discusses the challenges of implementing these compounds clinically, as well as potential alternatives to achieve this goal.

## 2. Antimicrobial Peptides

**Figure 2:**
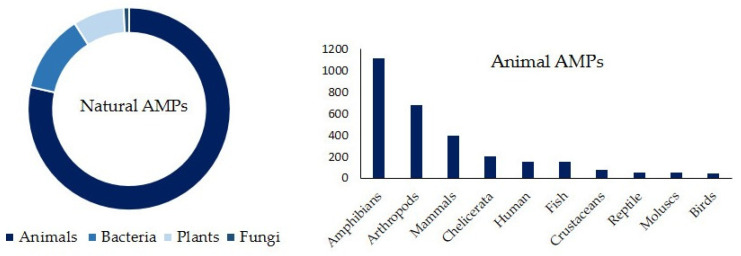
Distribution of Natural AMPs by Origin [21].

AMPs are endogenous compounds produced by various organisms, including humans, as well as both prokaryotic and eukaryotic organisms. In 1939, microbiologist René Dubos discovered the first AMP, gramicidin, which was isolated from a *Bacillus* strain that exhibited antipneumococcal activity in mice. Since then, many more AMPs have been discovered in a wide variety of organisms [20]. As of January 2025, the Antimicrobial Peptide Database (APD) contained over 5000 AMPs, approximately 3000 of which occur naturally. Most of these were isolated from eukaryotes, particularly animals (Figure 2) [21].

Most AMPs are produced in response to bacterial infections and can neutralize multiple microorganisms. It is now recognized that AMPs are a major component of the innate immune system of all living organisms [22,23,24].

Bactericidal AMPs, also called bacteriocins, produced by bacteria have been studied for their potential as food preservatives and antimicrobials. Traditionally, they have been credited with the fundamental role of destroying and limiting the growth of competing bacteria within the same ecological niche [25]. The production of antimicrobial substances, such as hydrogen peroxide, fatty acids, organic acids, ethanol, lytic enzymes, and antibiotics, is a well-known example of microbial antagonism. However, the exact function of bacteriocins in various natural environments remains unclear. Some authors have proposed that these compounds could play additional ecological roles, such as mediating cell lysis, releasing DNA for natural transformation processes, and interacting with regulatory networks in bacterial cells [26].

Biofilm formation is a characteristic of many infectious processes caused by bacteria and other microorganisms. These communities are formed when cells adhere to a surface and produce an extracellular polymeric matrix, which is primarily composed of polysaccharides, proteins, and DNA. This matrix protects the bacteria from environmental stressors and makes them resistant to many antibiotics. Current research has demonstrated the great potential of AMPs as antimicrobial and anti-biofilm agents [27,28]. Additionally, AMPs with anti-inflammatory, anti-cancer, and immune-modulating properties have been identified [29,30,31]. These additional roles have prompted a shift in terminology, replacing AMPs with the broader term “host defense peptides” (HDPs) to capture these molecules’ multifunctional nature [32].

### 2.1. Classification of Naturally Occurring AMPs

**Figure 3:**
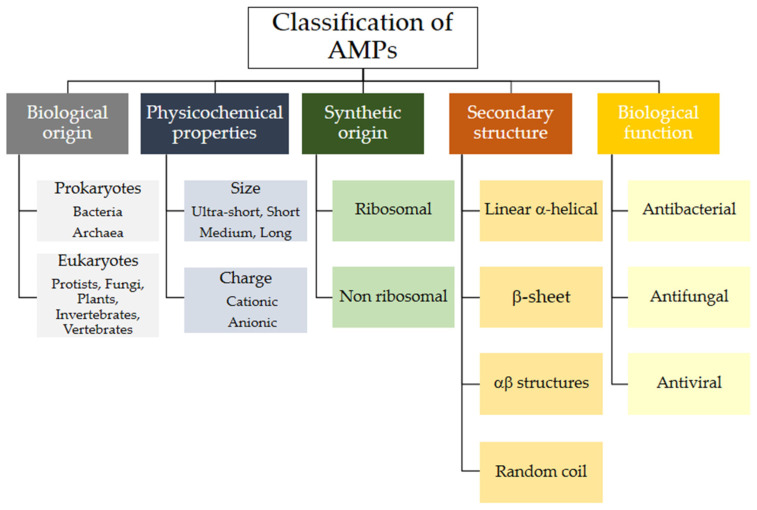
Classification of AMPs according to some of the different criteria used by APD3 [21].

Various criteria can be used to classify natural AMPs. The APD classifies them by biological origin, physical and chemical properties, biosynthetic origin, biological function, types of covalent bonds formed, three-dimensional structure, biological target, and mechanism of action (see Figure 3). Below is a brief review of some of these aspects. The following sections will classify AMPs according to their origin, whether biological or synthetic [33].

AMPs share many characteristics. They are small proteins consisting of fewer than 100 amino acids (aa), typically between 20 and 50, with an average of 33.26. They have a positive net charge ranging from +2 to +11, averaging 3.32. AMPs are composed of multiple arginine (R), lysine (K), and/or histidine (H) residues, and they have a high proportion of hydrophobic residues (approximately 50%). An appropriate combination of cationic and hydrophobic aa is essential in AMPs. Their structure is amphipathic, meaning they have both hydrophobic and hydrophilic regions. This allows them to solubilize in aqueous environments and provides structural flexibility. Depending on their size, AMPs are classified as ultra-short (2–10 aa), short (10–24 aa), medium (25–50 aa), or long (50–100 aa). Those with more than 100 aa are considered antimicrobial proteins. [34].

It is important to note the significance of the high concentrations of Tryptophan (W) and R in the AMP composition. As a charged amino acid, R forms ionic and hydrogen bonds with the abundant anionic components of bacterial membranes, which promotes initial interactions between peptides and bacteria. W is a hydrophobic amino acid that affects the interfacial region of the lipid bilayer, helping to anchor the peptide within it. The presence of R and W amino acids enables cation-π interactions within the AMP. These interactions contribute to the AMP’s deeper insertion into the lipid membrane and stabilize its structure [35,36].

Some AMPs have been shown to be especially rich in a single amino acid. Proline-rich AMPs (PrAMPs), for example, can enter bacterial cytoplasms through the inner membrane transporter SbmA. Once inside, PrAMPs target ribosomes and interfere with protein synthesis. They do so by blocking the binding of aminoacyl-tRNA to the active site of peptidyltransferase and trapping ribosomal release factors. Though different PrAMPs lack high sequence similarity, they contain short motifs with repeated proline and arginine residues (e.g., -PPXR- in Bac5 and -PRPX- in Bac7). PrAMPs primarily kill Gram-positive bacteria [34,37]. Histidine-rich AMPs, such as histatin peptides, are characterized by their ability to permeate cell membranes [38]. Glycine-rich AMPs, such as attacking and dipteric peptides, are widely distributed in nature. These peptides contain 14–22% glycine residues, which significantly affect the tertiary structure of the peptide chain [32].

It is worth mentioning the existence of an uncommon class of AMPs: anionic AMPs. These compounds have been identified in vertebrates, invertebrates, and plants. They contain several aspartic and glutamic acid residues, which give them an overall negative charge ranging from −1 to −7. Unlike cationic AMPs, anionic AMPs use metal ions to form cationic salt bridges with the negatively charged components of microbial membranes, facilitating their entry into cells. Once inside the cell, anionic AMPs can either bind to ribosomes or inhibit ribonuclease activity [20]. In scientific literature, the term “antibacterial peptides” is usually only applied to cationic types, which is what we will refer to in this review.

#### 2.1.1. Classification of AMPs by Biosynthetic Origin

Based on this criterion, two types of AMPs are distinguished: ribosomally synthesized and post-translationally modified peptides and non-ribosomal peptides (NRPs). Ribosomal AMPs serve as the primary defense mechanism in prokaryotes, plants, and animals. NRPs are produced only by bacteria and fungi and are typically called polypeptide antibiotics. The term “AMP” usually refers to ribosomal antibiotics [29].

#### 2.1.2. Classification of AMPs by Structure

**Figure 4:**
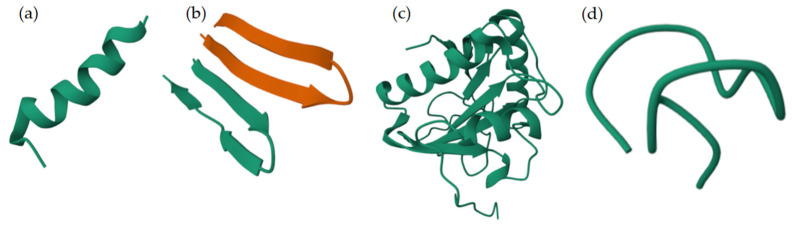
Structures of several AMPs taken from the PDB. (**a**) Antimicrobial peptide RP-1 bound to SDS micelles (18 aa, α-helix L2-R16) [43]; (**b**) Antimicrobial peptide Arenicin-2 dimer in DPC micelles (two chains with 21 aa every one; chain A: β-sheet formed by three β-strands C3-I10, V13-V15 and R18-C20, and a β-turn R11-G12 stabilized by a disulfide bridge C3-C20; chain B: β-sheet formed by two strands 2W-I10, V13-W20 and a β-turn R11-G12 stabilized by a disulfide bridge C3-C20. The dimer is stabilized by seven intermolecular hydrogen bonds) [44]; (**c**) *Drosophila* Peptidoglycan Recognition Protein (PGRP)-SA (180 aa, α-helix L16-G21, S49-E68, S121-Q238, S151-V153, G160-Q168; β-sheet: I14-K15, I35-H42, F78-I80, V86-E88, G106-F111, L141-A149) [45]; (**d**) Temporin L in solution (13 aa, unstructured) [46].

NMR spectroscopy in various media, including water, aqueous solutions of fluorinated alcohols (e.g., trifluoroethanol and hexafluoroisopropanol), and sodium dodecyl sulfate (SDS) micelles, which mimic the physicochemical characteristics of cell membranes, has been essential to studying the structure of AMPs [39]. Similarly, molecular dynamics simulations involving membranes, along with X-ray diffraction and circular dichroism spectroscopy, have provided valuable insights into the structure and function of the molecule [40]. Currently the Protein Data Bank (PDB) contains over 600 entries related to AMP structure [41]. According to Wang’s classification, AMPs can be divided into four groups based on their secondary structure elements: α-helical peptides, β-sheet peptides (including β-hairpins or loops with one disulfide bond and β-sheets with two or more disulfide bonds), peptides with a mixed α/β structure, and non-α peptides with an extended or random coil structure (Figure 4) [42].

Alpha-helical peptides are the most widely studied. They typically have an amidated C-terminus and a net charge ranging from +2 to +9. They also usually contain more than 50% hydrophobic aa. Their cationic and hydrophobic domains are arranged on opposite faces of the helix, facilitating interaction between AMPs and membranes. Alpha-helical peptides are unstructured in aqueous solution, but they adopt an amphipathic helical structure when in contact with membrane-mimicking environments. In this structure, the distance between two adjacent amino acids is approximately 0.15 nm, and the angle between them is 100°. The presence of α-helical motifs is key to promoting interactions between peptides and target membranes, which allows for their disruption. Disturbing the α-helical structure through aa substitutions significantly reduces antibacterial activity. The helical structure of AMPs is also associated with hemolytic activity and toxicity in mammalian cells [47,48]. A notable feature of these peptides is their potential to interact with bacterial lipopolysaccharides (LPS). This interaction induces conformational changes that affect membrane permeabilization and proper passage to the cytosol [20].

AMPs that exhibit a β-sheet conformation consist of at least one pair of β-chains linked by disulfide bonds. These bonds, along with salt bridges and head-to-tail cyclization, stabilize the structure. These AMPs have a more stable structure and do not undergo significant conformational changes when interacting with biological membranes. AMPs contain spatially segregated β-chains as polar and nonpolar domains, giving them an amphipathic character. In the case of β-hairpin AMPs, the characteristic element is a hairpin stabilized by an intrachain disulfide bridge [47].

Some AMPs adopt a mixed α-helix/β-sheet structure stabilized by three or four disulfide bridges. The cysteine-stabilized α/β (CSαβ) structural motif consists of one α-helix and one or two antiparallel β-sheets. It was initially identified in insect antibacterial defensins and scorpion neurotoxins. This motif is prevalent in plant and insect AMPs. These AMPs exhibit an amphipathic structure in which positively charged residues are typically found in the helix, while the β-sheet consists of hydrophobic amino acid residues. Most AMPs exist in an unstructured state in aqueous solutions and undergo conformational changes, adopting a well-defined structure depending on environmental conditions [47].

#### 2.1.3. Classification of AMPs According to Their Biological Function

The ADP3 database categorizes peptides by their biological function into 18 groups. However, regarding AMPs, three groups can be distinguished: Antibacterial peptides (ABPs), which represent a large proportion of AMPs, exhibit broad inhibitory effects against common pathogenic bacteria. Many natural and synthetic ABPs demonstrate good inhibitory activity against both Gram-positive and Gram-negative bacteria. Antifungal peptides (AFPs) are a subclass of AMPs that address fungal infections with increased drug resistance. Many AFPs demonstrate excellent antifungal activity against common pathogenic fungi, such as *Aspergillus* and *Candida albicans*, which are important in clinical medicine. They are also effective against yeasts and filamentous fungi, such as *Aspergillus flavus*, as well as molds in food and agriculture. A multitude of synthetic peptides with good antifungal activity have also been described. Finally, antiviral peptides (AVPs) exhibit a potent killing effect on viruses. They primarily accomplish this by inhibiting adhesion and fusion with cell membranes, destroying envelopes, and/or inhibiting replication. This group includes anti-HIV peptides [34].

### 2.2. Mechanism of Action of AMPs

**Figure 5:**
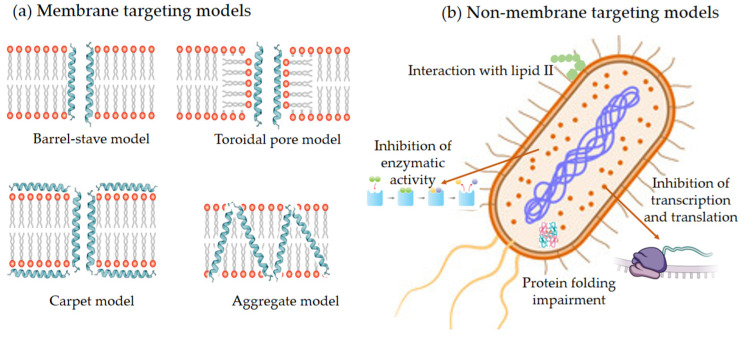
Main mechanisms of action of AMPs. There are two types of strategies: membrane-directed (**a**) and non-membrane-directed (**b**). The former involves interaction with and lysis of the membrane, either through the formation of transmembrane pores (barrel-stave and toroidal-pore models) or without (carpet and aggregate models). The text provides more details about each of these mechanisms. Among the non-membrane-directed mechanisms, one can distinguish between cell wall targeting, whereby AMP binds to components or precursors of this structure (e.g., lipid II), and intracellular targeting, whereby AMP integration results in negative effects on essential processes, such as replication, transcription, translation, enzymatic activities, and protein folding.

Cationic AMPs exert their activity by interacting with microbial cell membranes, and this interaction is strongly influenced by the membranes’ lipid composition. Because bacterial membranes are the primary targets of AMPs, bacteria find it more difficult to develop resistance to these drugs than to conventional antibiotics. As previously mentioned, electrostatic interactions between AMPs and negatively charged microbial surfaces are crucial. Teichoic acids in the cell wall of Gram-positive bacteria and LPS in the outer membrane of Gram-negative bacteria give their surfaces a negative charge, which strengthens the interaction with AMPs. In contrast, the outer layer of eukaryotic membranes is neutral at physiological pH because it consists mainly of phosphatidylcholine and sphingomyelin. Thus, this interaction is not favored [20]. Numerous articles and reviews in the literature describe the mechanisms of action of AMPs [47,48,49,50,51,52]. In general, two types of strategies can be distinguished (Figure 5): (a) membrane-directed mechanisms and (b) non-membrane-directed mechanisms. Type (a) proposes that the bactericidal effect of AMPs results from their interaction with the membrane, increasing its permeability, and ultimately lysing the membrane and releasing cellular contents. Depending on whether or not pores are present in the membrane following this mechanism, we can differentiate between transmembrane pore models (barrel-stave model and toroidal-pore model) and non-porous models (carpet model and aggregate model). According to the barrel-stave model, AMPs with an amphiphilic structure aggregate on the bacterial cell membrane as a barrel-shaped plate around its axis. This forms transmembrane pores that allow the filtration of cellular contents, ultimately leading to cell death. According to the toroidal-pore model, AMPs aggregate in the cell membrane to form a pore. They act as the outer ring, and the phospholipid head acts as the inner ring. This ultimately leads to the leakage of intracellular material. In the carpet model, AMPs spread across the cell membrane surface. When they reach a certain concentration, they destroy the membrane’s structure, causing leakage of cellular contents. Those acting according to the aggregate model insert themselves into the cell membrane through hydrophobic interactions, rupturing it and causing cell death in a manner similar to how detergent dissolves fat [52]. Two types have also been identified in non-membrane-targeted mechanisms (Figure 5b): cell wall-targeting and intracellular targeting. In the former, Lipid II is the key element to which AMPs bind by recognizing its structural domain. This creates a spatial barrier that obstructs cell wall synthesis. This interaction causes membrane perforation, resulting in morphological abnormalities and, ultimately, bacterial lysis. Some AMPs, such as nisin, exhibit “dual-mechanism synergistic sterilization”. The *N*-terminal ring of this AMP binds specifically to the pyrophosphate group of Lipid II, which effectively inhibits transglycosylase activity. Meanwhile, the C-terminus inserts into the cell membrane to form pores. Intracellular targeting mechanisms involve AMP penetration into the cytoplasm and interaction with various intracellular targets. These interactions interfere with the essential cellular activities necessary for achieving biological effects, including DNA and RNA synthesis, protein folding, and enzymatic activity [20,48,52,53,54,55].

In the following sections, these aspects will be reviewed again for the different types of AMPs classified according to their biological and synthetic origin (see Figure 2 and Figure 3).

## 3. Antimicrobial Peptides of Animal Origin

In general, mammalian AMPs are classified into three major families: defensins, cathelicidins, and histatins. These types of AMPs are also found in other animals, including birds and fish [29]. Mammalian-derived peptides act as HDPs and exhibit broad-spectrum antimicrobial activity against bacteria, viruses, and fungi. These peptides protect humans from infections. Some researchers suggest that promoting the innate immune system through HDPs is the primary mechanism for eliminating infectious agents early on. Most human HDPs are produced by epithelial, inflammatory, and immune cells in response to microbial invasion. Several studies of the non-microbicidal properties of HDPs have examined their impact on immune cells, including their capacity to recruit leukocytes. This presents significant therapeutic potential. Kim et al. (2023) [56] provide examples of this in their review. For instance, cathelicidin LL37 exhibits antimicrobial and antiviral activities by enhancing the early response of neutrophils rather than directly killing microbes, thereby acting in a protective manner. Additionally, β-defensin 2 (HBD-2) contributes to antitumor natural killer cell and beneficial T cell responses in murine models. It is also important to note that HDPs can modulate other immune cells, such as macrophages and mast cells, and indirectly recruit leukocytes via chemokine release [56,57].

The expression levels of these compounds vary depending on the growth stage. For example, cathelicidin LL-37 is predominant in the skin of newborns, while human beta-defensin 2 (hBD-2) is prevalent in the skin of elderly individuals. HDPs are found in various parts of the body, including the skin, eyes, ears, mouth, respiratory tract, lungs, intestines, and urethra. From a structural perspective, HDPs are characterized by stabilization through disulfide bonds and a cationic nature, similar to other AMPs.

Defensins were the first AMPs isolated from animals. The first defensin was found in rabbit lung macrophages in 1980 [58]. These proteins range in size from 3 to 5 kilodaltons (kDa) and contain three to six disulfide bridges that contribute to the stabilization of their beta-sheet secondary structure. The position of these bridges is conserved and determines their class.

α- and β-defensins are found in humans, as well as in the granules of immune cells, epithelial tissue, body fluids, and the mucosal surfaces of other vertebrate species. The third class of defensins, θ-defensins, are characterized by a cyclic peptide backbone connected by three parallel disulfide bridges. This structure’s stability likely enhances its antimicrobial activity. For example, in the case of human θ-defensin-1 (retrocycline-1), disulfide bridges and circularity have been reported to enhance receptor binding activity and inhibit HIV-1 entry [48]. All types of defensins must undergo proteolytic digestion to be activated [40,59,60,61].

The cathelicidin family comprises small cationic AMPs, also distributed in many vertebrates. This family includes approximately 30 peptides in mammals. These peptides are synthesized and stored in neutrophils and macrophages and are part of the innate immune system. Many cathelicidins have an unstructured conformation in aqueous solutions, yet they adopt amphipathic α-helical structures in environments that mimic biological membranes. This group also includes small peptides with β-hairpin structures stabilized by disulfide bonds. Similarly to defensins, cathelicidins are inactive propeptides with a conserved N-terminal domain in the precursor protein known as cathelin. This domain is proteolytically cleaved to generate the active peptides. Thus, these molecules consist of the C-terminal fragment, which exhibits greater variation in amino acid sequences, structures, and lengths. Their mode of action is based on their ability to interact with microbial membranes due to their amphipathic nature, which directly relates to their microbicidal effects. Furthermore, these molecules can cross the cell surface and bind to intracellular components, including protein complexes, ribosomes, DNA, and RNA. This affects multiple processes, including protein synthesis and folding, peptidoglycan biosynthesis, respiration, and the detoxification of reactive oxygen species. Cathelicidins collectively play various important roles in the host. They contribute to the direct elimination of pathogens, as well as to the modulation of the immune response, wound healing, and tumor development [34,62].

Histatins are low-molecular-weight peptides (approximately 3–4 kDa) containing a high number of histidine residues. They are part of the functional family of cationic peptides and exhibit weak amphipathic properties. Initially isolated from human parotid salivary glands, they are found in humans and higher primates. They are primarily synthesized and secreted by acini in serous glands, including the parotid, submandibular, and sublingual glands. Twenty-six histatins have been identified in human saliva, with histatins 1, 3, and 5 constituting over 80% of the total concentration of these proteins. They have linear structures and differ primarily in the number of aa: 38 for histatin 1, 32 for histatin 3, and 24 for histatin 5. Each contains seven histidine residues. In this family of proteins, histatins 1 and 3 are full-length products encoded by genes located on chromosome 4. Through proteolysis, histatin 1 produces histatin 2, and histatin 3 produces the other subtypes. Histatin 5 is derived from histatin 3 and contains an *N*-terminus that makes it highly reactive with metal ions. Histatins exhibit antimicrobial and cell-stimulating properties [63,64]. The aa histidine is essential to the bactericidal and fungicidal activities of these peptides. Deletion of this aa, particularly at position 5, significantly reduces histatin’s antifungal activity against common yeasts such as *Candida glabrata*, *Candida krusei*, *Cryptococcus neoformans*, and *Saccharomyces cerevisiae*. In this case, a distinct mechanism of action has been proposed. Histatins destroy the phospholipid membrane of target bacteria by interacting electrostatically with the bacterial cell membrane. The histatins then aggregate and interact with the lipid bilayer, resulting in the expansion of the outer leaflet and the local thinning of the membrane. This process is independent of the peptide’s chiral conformation. In contrast, antifungal activity results from a molecular mechanism involving the initial recognition and binding of a specific protein receptor by the peptide. This is followed by internalization and interaction with intracellular targets. Of the histatins, histatin 5 exhibits the most potent antimicrobial activity. Therefore, most histatin research has focused on this peptide [64,65,66].

Amphibian AMPs play an important role in protecting against pathogens. Many species of anuran amphibians (frogs and toads) belong to the *Alytidae*, *Bombinatoridae*, *Hylidae*, *Hyperoliidae*, *Leiopelmatidae*, *Leptodactylidae*, *Myobatrachidae*, *Pipidae*, and *Ranidae* families, secreting cytotoxic peptides in high concentrations. These peptides exhibit broad-spectrum antibacterial and antifungal properties and can permeabilize mammalian cells. These multifunctional components display cytokine-mediated immunomodulatory properties, as well as anti-cancer, anti-viral, chemotactic, and insulin-releasing activities. Since not all anurans produce host-defense peptides in their skin secretions, this sporadic distribution suggests that producing these peptides may confer an evolutionary advantage, though they are not necessary for survival [67].

The size of frog skin HDPs ranges from 8 to 63 amino acid residues. A comparison of their amino acid sequences reveals an absence of domains associated with biological activity. These peptides are generally cationic, with a charge ranging from +2 to +6 at pH 7, and they contain approximately 50% hydrophobic amino acids. The *Pipidae* family, for example, which includes the species *Xenopus*, *Silurana*, *Hymenochirus*, and *Pseudhymenochirus*, has a high concentration of AMPs. While these peptides vary in size and sequence, they generally adopt an α-helix conformation under membrane-mimicking conditions [67].

The venom of various animals, including snakes, bees, spiders, scorpions, and frogs, is a complex mixture of enzymatic and non-enzymatic components that perform specific pathophysiological functions. These venoms serve as defense mechanisms or are used to immobilize and digest prey. Peptide toxins isolated from these venoms primarily target ion channels, membrane receptors, and components of the hemostatic system with high selectivity and affinity. Snake venoms, for example, have been used as medical tools for thousands of years, particularly in traditional Chinese medicine. Consequently, snake venoms can be considered miniature drug libraries, with each toxin being pharmacologically active. Some commonly used drugs, such as captopril, are derived from compounds found in snake venom [68].

Scorpion venom is also of great interest to researchers. Over millions of years, the venom sac of scorpions has evolved to become a rich reservoir of proteins and non-protein macromolecules. Some of the compounds found in scorpion venom, such as certain peptides and proteins, have shown promise in medical research for potential applications [69].

Spider venom is an intricate mixture of biologically active peptides. Studies examining the venom composition of *Lycosidae* spiders have revealed neurotoxic peptides with multiple disulfide bridges and antimicrobial peptides without cysteine residues. Lycosin-I, which was isolated from the venom of *Lycosa singoriensis*, demonstrates significant antibacterial properties and has potential applications in antitumor therapies. Additionally, 52 antimicrobial peptides were identified in the venom of *Lycosa sinensis*. LS-AMP-E1 and LS-AMP-F1, derived from the wolf spider *L. sinensis*, can inhibit multidrug-resistant strain growth and sterilize bacteria when used with conventional antibiotics. Currently, there are over 15 venom-derived peptides undergoing clinical trials, including four AMPs [68,70].

In insects, AMPs are primarily synthesized in the fat bodies and blood cells. Two well-known AMP families are the cecropins, found in guppies, silkworms, bees, and *Drosophila*, and the defensins, which exhibit antimicrobial activity against fungi and bacteria. The amount of AMPs varies considerably between species, and they can exhibit diverse secondary structures. For instance, cecropins have α-helical structures, whereas insect defensins form β-sheet and α-helix/β-sheet mixed structures stabilized by three or four disulfide bridges (CSαβ) [47]. Other AMPs, such as drosocin and lebocin, have extended structures. Like other PrAMPs, these proline-rich AMPs can enter cells and interfere with intracellular activities, such as protein synthesis [29,34,67,71,72,73]. Table S1 in the Supplementary Material provides examples of different types of animal-derived AMPs.

## 4. Plant AMPs

Most plant AMPs are positively charged at physiological pH and have molecular masses ranging from 2 to 10 kDa. Their sequences are highly variable and contain 4 to 12 cysteine residues that form disulfide bonds. These bonds stabilize the tertiary and quaternary structures of AMPs, making them exceptionally resistant to chemical, thermal, and enzymatic degradation. AMPs production occurs in various plant tissues, including leaves, roots, seeds, flowers, and stems. This indicates that they are immediately available at any infection site. Defense mediated by these AMPs is achieved not only by the presence of a single AMP at the infected site but also by a mixture of peptides with different characteristics and mechanisms of action. In fact, a single plant species can contain several AMPs. This defense strategy is effective, as evidenced by the increased tolerance to pathogen attack exhibited by transgenic plants that overexpress AMP-encoding genes. Plant AMPs play a significant role not only in defending plants against pathogens, but also in affecting plant growth and development. The smallest known AMP, composed of seven aa, was isolated from *Jatropha curcas*. Plant AMPs are difficult to classify due to their diverse functions, structures, expression patterns, and specific targets. They are typically differentiated based on sequence similarity, cysteine motifs that determine unique disulfide bond patterns, and tertiary structure folding. Examples of these AMPs include thionins, defensins, hevein-like peptides, knottin-like peptides (linear and cyclic), α-hairpin family lipid transfer proteins, snackins, and cyclotides. Plant AMPs can tolerate hypervariable sequences while maintaining a conserved scaffold via disulfide bonds. This diversity allows them to recognize different targets through sequence variation in non-cysteine residues [74,75].

## 5. AMPs Produced by Bacteria

### 5.1. Bacteriocins or Ribosomal AMPs

The first bacteriocin, *colicin*, was discovered in *E. coli* in 1925. Bacteriocins are a highly diverse class of bactericidal peptides or proteins synthesized by bacteria and archaea via ribosomal processes. Most bacterial species, including both Gram-positive and Gram-negative ones, can produce at least one bacteriocin. These compounds exhibit bacteriostatic or bactericidal activity and a specific immunity mechanism toward closely related bacterial strains. In rare cases, they can affect a broader range of unrelated bacterial groups. Like other AMPs, almost all bacteriocins are small cationic molecules with hydrophobic or amphiphilic characteristics. Initially produced as linear peptides, bacteriocins undergo several post-translational modifications (epimerization, heterocyclization, and macrocyclization) after release into the cytoplasm, which allows them to adapt to their specific function [76,77,78,79].

Bacteriocins derived from lactic acid bacteria (LAB) are of great interest because they are produced by GRAS (Generally Recognized as Safe) organisms and are classified as QPS (Qualified Presumption of Safety). This means that they are considered safe by the United States Food and Drug Administration (USFDA). These molecules may or may not undergo post-translational modification (PTM) processes, and they are heat-stable yet sensitive to digestive proteases. They exhibit significant diversity in terms of length, genetic origin, biochemical characteristics, molecular mass, cellular receptors, and interaction with the immune system. Their inhibitory mechanism is thought to involve membrane permeabilization through pore formation. However, other modes of action have been suggested, including the inhibition of cell wall biosynthesis and interference with metabolic pathways. Bacteriocins exhibit high inhibitory activity at concentrations in the nanomolar range and have a relatively narrow antimicrobial activity spectrum. The most studied LAB bacteriocin is nisin A, which is produced by *Lactococcus lactis* strains and is currently used as a preservative in the food industry. LAB bacteriocins have been shown to protect the gastrointestinal tract by eliminating pathogens or preventing bacterial colonization of the intestine. They are active against intestinal pathogens, including *Listeria monocytogenes*, *Salmonella enteritidis*, *Clostridium difficile*, *S. aureus*, and VRE. Some have been shown to effectively treat multidrug-resistant (MDR) infections [79,80,81].

Based on their chemical structure, amino acid modifications, enzyme sensitivity, size, thermostability, and mechanisms of action, AMPs from *Gram-positive bacteria* have been classified into four categories: (I) lantibiotics, (II) non-lantibiotics, (III) large bacteriocins, and (IV) uniquely structured bacteriocins [61].

Class I bacteriocins, also known as lantibiotics, are small peptides (less than 5 kD, 19–38 aa) that are stable under heat, a wide pH range, and proteolysis. They incorporate unusual amino acids, such as lanthionine and β-methyllanthionine, through post-translational modifications. They are primarily active against Gram-positive bacteria. Lantibiotics are further divided into subclasses. Subclass Ia consists of positively charged, elongated peptides (e.g., nisin, epidermin, gallidermin, and Pep5), which form pores in bacterial membranes. This allows for the efflux of small molecules, dissipation of the membrane potential, and ultimately, the arrest of cellular biosynthesis. Nisin and epidermin have an additional mode of action that interferes with cell wall synthesis by inhibiting transglycosylation of peptidoglycan. Subclass Ib consists of negatively charged, globular, and inflexible peptides (e.g., lacticin, cytolysin, and salivaricin) which inhibit crucial enzymes in target bacteria. Class II bacteriocins (non-lantibiotics) undergo limited post-translational modification and therefore do not contain lanthionine or unusual amino acids. These small peptides (less than 10 kDa, 30–60 aa) have one or more disulfide bonds in their structure, and they are thermostable. They act as pore-forming, membrane-destabilizing bacteriocins that increase membrane permeability. Class II bacteriocins can be classified into four subclasses. The largest group is IIa (pediocin-like), which contains the *N*-terminal motif YGNGV and is highly effective against *L. monocytogenes*. Subclass IIb requires the combined action of two peptides to be effective. Subclass IIc has a cyclic structure formed by covalent bonds of the carboxyl and amino groups of the terminal residues. Finally, subclass IId consists of various types of linear peptides. Class III bacteriocins, also known as bacteriolysins, consist of large peptides (more than 30 kDa) that are heat labile and have an active -SH group. Examples include zoocin A, lysostaphin, enterolysin A, and heleveticin M, J, and V, which exhibit endopeptidase-like activity against peptidoglycan and causes cell wall disruption. Class IV AMPs, such as plantaricin S, leuconocin S, lactocin 27, and pediocin SJ-1, have a unique structure. They contain amino acids, lipids, and carbohydrates, making them susceptible to numerous lipolytic and glycolytic enzymes. They disrupt cell membranes [61,82].

Regarding bacteriocins isolated from *Gram-negative bacteria*, the best-known correspond to those of *E. coli*, although other species, such as *Klebsiella* spp. and *Pseudomonas* spp., also synthesize AMPs. These bacteriocins have a limited spectrum of activity against Gram-negative organisms and can be classified into four categories: colicins, colicin-like bacteriocins, microcins, and phage-tailed bacteriocins [61].

Colicins (MW more than 10 kDa) are predominantly produced by *E. coli*. They bind to specific cell surface receptors before translocating across the outer membrane, periplasm, and inner membrane into the cytoplasm. Colicin production kills susceptible neighboring cells and the producing cells. Colicins can be divided into four types based on their mode of action: (i) formation of channels in the cytoplasmic membrane, (ii) degradation of DNA, (iii) degradation of RNA (either ribosomal or transfer), and (iv) inhibition of murein and lipopolysaccharide biosynthesis. Colicin-like bacteriocins have similar structures and functions, but they are produced by different species, including *P. aeruginosa* (S-type pyocins) and *Klebsiella* spp. (klebicins). S-type pyocins are protease-sensitive and induce cell death by cleaving DNA or RNA or by forming channels. Klebicins act through endonuclease activity, pore formation, and/or degradation of peptidoglycan in the cell wall. Microcins are small (<10 kDa) peptides produced by *Enterobacteriaceae* that are active against phylogenetically related species. They are classified into two subclasses. Subclass I microcins (MW less than 5 kDa, including microcins B17, C7, D93, and J25) undergo extensive PTM of the backbone, whereas subclass II (MW 5 to 10 kDa, including microcins E492, V, L, and H47) are either lightly modified or unmodified. Microcins have many modes of action, depending on their diverse cellular targets. For example, E492 forms pores in the bacterial membrane and disrupts the membrane potential, and B17 interferes with replication by targeting DNA gyrase. J25 appears to have a dual mechanism of action involving interference with RNA polymerase and membrane disruption. Phage tail bacteriocins are high-molecular-mass cylindrical peptides that are named for their close resemblance to the structure of the phage tail. They can be divided into two main classes: R-type, which are related to the contractile tails of *Myoviridae phages*, and F-type, which are related to the tails of *Siphoviridae phages*. R-type bacteriocins initially bind to cell surface receptors. Then, the sheath contracts and forces the inner core into the cell envelope. This process creates a channel through which ions flow, which uncouples cellular ionic gradients and respiration. This results in rapid cell death. Additionally, they interfere with oxygen uptake and macromolecule synthesis. Unlike R-type bacteriocins, F-type bacteriocins lack a contractile mechanism. However, they are thought to form a channel similar to that of R-type bacteriocins in the inner membrane, resulting in death by a similar mechanism [61]. Table S2 in the Supplementary Material lists several examples of bacterial AMPs of ribosomal origin.

### 5.2. Non-Ribosomal AMPs

Non-ribosomal antimicrobial peptides (NRAMPs) are only found in bacteria and fungi. These microbial secondary metabolites exhibit enormous structural diversity and a wide range of biological activities that are useful for medical and agroecological applications. NRAMPs are produced by non-ribosomal peptide synthetases (NRPSs), which are more specialized for specific functions. NRPSs are large, multi-modular enzyme complexes that assemble specific peptide products by sequentially condensing amino acids and amino acid-like substances, independently of the ribosome. NRPSs range in size from 100 to over 1600 kDa and can incorporate non-canonical and non-proteinogenic amino acids, such as α-, β-, or γ-amino acids, D-amino acids and other analogs. NRPSs can also incorporate various hydroxy or ketocarboxylic acids. The *N*-terminus of NRAMPs is often modified by fatty acids, heterocyclic compounds, glycosylated structures, and phosphorylation. NRAMPs are short, linear, cyclic, or branched cyclic peptides. Linear NRAMPs have antimicrobial, insecticidal, antiviral, and anti-cancer properties. Cyclic NRAMPs are obtained by macrolactonization or macrolactamization without additional PTMs. Unlike ribosomal homodetic cyclic peptides, which only exhibit a head-to-tail topology, the non-ribosomal pathway produces both head-to-tail and head-to-side chain versions. NRAMPs also exhibit different bond types. For instance, disulfide bridges, lanthionine thioethers, and one-carbon thioethers are prevalent in the ribosomal pathway. Conversely, depsi bonds, biaryl bonds, and aryl ethers are prevalent in the non-ribosomal pathway [82,83,84,85]. The review by Monaim et al. (2019) [86] provides a clear classification of the two types of AMPs, their production sources, and their main structural differences. *Tridecaptins* are an example of NRPs. They have a linear structure and are selective against Gram-negative bacteria. These compounds are naturally produced by *Bacillus* and *Paenibacillus* species. Tridecaptins are cationic lipopeptides consisting of 13 aa (including non-proteinogenic residues) and a chiral lipid tail. For example, TriA1 has a (3*R*,6*S*)-3-hydroxy-6-methyloctanoyl lipid tail. The first three tridecaptins were discovered in 1978 and they differ in their aa composition and lipid tail structure. They act by binding selectively to the precursor lipid of peptidoglycan II in Gram-negative bacteria, thereby altering the proton-motive force. Several synthetic analogs with improved antimicrobial activity and stability have been described. Additionally, it has been demonstrated that non-acylated tridecaptins can act synergistically with clinically relevant antibiotics by sensitizing the outer membrane [85].

Polymyxins are a class of cyclic lipopeptides produced by the Gram-positive bacterium *Paenibacillus polymyxa*. They are active specifically against Gram-negative bacteria. More than thirty natural and synthetic polymyxins have been documented in the literature. Two of these (polymyxin B and colistin/polymyxin E) are used in clinical settings. Polymyxins are characterized structurally by a positively charged γ-diaminobutyric acid-rich cyclic heptapeptide headgroup and a long hydrophobic acyl tail that is linked to the headgroup by a positively charged tripeptide bond. Polymyxins bind to the negatively charged lipid A headgroups of LPS in Gram-negative bacterial membranes through electrostatic interactions. Their cationic charge also displaces the normally occurring divalent Ca^2+^ and Mg^2+^ ions that bind adjacent LPS molecules in the bacterial outer membrane. This disruption increases membrane permeability. Once the outer membrane is compromised, polymyxins can enter the periplasm and bind to LPS molecules on the cytoplasmic membrane. This disrupts the cytoplasmic membrane’s structural integrity, ultimately causing cell death. Additional mechanisms of action upon cell entry have been proposed, including inhibiting respiratory enzymes, binding to ribosomes, and disrupting cell division [87,88].

Other non-ribosomal lipopeptides of bacterial origin include brevicidins, laterocidins, and relacidins. These compounds are all active against Gram-negative pathogens [89].

## 6. Fungal AMPs

There are two main types of fungal AMPs: defensins and peptaibols. Fungal defensins are called defensin-like peptides (DLPs) because of their high sequence and structural similarities. The first characterized fungal defensin was plectasin, found in the species *Pseudoplectania nigrella*. Plectasin belongs to the ribosomal peptide family and exhibits activity against predominantly Gram-positive bacteria, including *Streptococcus pyogenes*, *Corynebacterium jeikeium*, *Corynebacterium diphtheriae*, and *S. aureus*. Plectasin is structurally similar to defensins found in plants and insects. It contains a core structural motif consisting of a cysteine-stabilized α/β fold. Many defensins are thought to act by disrupting the microbial cytoplasmic membrane. However, plectasin acts by binding directly to lipid II, the precursor of the bacterial cell wall, thereby inhibiting cell wall biosynthesis. Other fungal defensins include copsin, produced by *Coprinopsis cinerea*, and mycasin, from *Microsporum canis* [61,90].

Peptaibols are short peptides consisting of 5–21 amino acids that are generated from the fungal NRPS pathway. To date, more than 1000 peptaibols produced by approximately 30 genera of filamentous fungi have been reported, most of which belong to the order *Hypocreales*. Peptaibols exhibit various properties, including antibacterial, antifungal, anti-cancer, immunosuppressive, anti-mycoplasma, anti-trypanosome, and wound-healing properties. They contain a high proportion of non-proteinogenic amino acids, such as α-aminoisobutyric acid (Aib), which accounts for 14–56% of their composition. They also have an acylated *N*-terminal residue and an aminoalcohol (e.g., phenylalaninol or leucenol) attached to the C-terminal residue. Due to the conformational constraints imposed by the abundance of Aib, peptaibols have a helical structure. Peptaibols are classified as follows: Long-sequence peptaibols (18 to 21 aa, typically with a proline and glutamine residue located centrally near both ends); Short sequence peptaiboles (11 to 16 aa, with several Aib-Pro motifs and typically Ac-Aib-Asn- or Ac-Aib-Gln- as the *N*-terminus); and Ultrashort lipopeptaiboles (7 to 11 as, with a high glycine content and *N*-terminal amino acids acylated by a C8 to C15 fatty acid). Longer peptaibols can form helical structures that oligomerize and create ion channels in the membrane. The action of short- and medium-chain peptaibols is more complex and likely results from a combination of membrane disruption and effects on different molecular targets. Examples of membrane disruption include forming transmembrane channels through helical bundles within the bilayer or via a barrel-stave mechanism. Alamethicin, the most studied peptaibol, was isolated from *Trichoderma viride*. It is active against both Gram-positive bacteria (e.g., *Enterococcus faecalis*, *Staphylococcus hemolyticus*, *S. aureus*, and *Streptococcus viridans*) and Gram-negative bacteria (e.g., *E. coli*, *K. pneumoniae*, *Proteus vulgaris*, and *P. aeruginosa*) as well as fungi. Other peptaibols include trichogin GA IV and tricholongin B from *Trichoderma longibrachiatum*; saturnisporin SA (SA II and SA IV) from *Trichoderma saturnisporum*; and silucine from *Rhizomucor pusillus*. Table S3 (in the Supplementary Material) lists various examples of non-ribosomal AMPs from bacteria and fungi [61,91,92,93].

## 7. Bacteriophage AMPs

Bacteriophages are viruses that infect bacteria. Active against both Gram-positive and Gram-negative bacteria, they were first used during World War II, before antibiotics were introduced. For instance, preparations of pathogen-specific bacteriophages (e.g., *P. aeruginosa*, *S. aureus*, *E. coli*, and *Klebsiella* spp.) were applied directly to extensive infections caused by war wounds. Many phage proteins, including endolysins, virion-associated peptidoglycan hydrolases (VAPGHs), depolymerases, and holins, exhibit antibacterial activity [61,94].

Phage-encoded lytic factors, also known as lysins, are enzymes that break down the bacterial cell wall by digesting peptidoglycan. This creates holes in the cell wall through which phage progeny can escape. They range in size from 25 to 40 kDa. Due to their unique properties, lysins are considered an alternative to or complement for antibiotics. These properties include: (i) rapid bactericidal activity due to action directed at the highly conserved bacterial peptidoglycan, (ii) synergy with cell-wall-inhibiting antibiotics, (iii) antibiofilm activity, and (iv) stability and thermostability. Examples include PK34 and LysAB2 P3, which are active against *Mycobacterium tuberculosis* and *A. baumannii*, respectively. The phage lysin PlyV12 exhibits broad bactericidal activity against *Enterococci* and other Gram-positive organisms, including *S. pyogenes*, *S. aureus*, and group B *Streptococci*. Several studies have demonstrated the increased antibacterial and antibiofilm potency of endolysins when used with antibiotics [15,61].

Virion-associated peptidoglycan hydrolases (VAPGHs) are primarily encoded by double-stranded DNA phages. These enzymes consist of a cell wall-binding domain at the C-terminus and one or more catalytic domains at the *N*-terminus. These enzymes exhibit bactericidal activity and thermostability. VAPGHs bind to bacterial cells through specific receptors on their surfaces. Local hydrolysis of the cell wall allows for the injection of phage genetic material. VAPGHs are classified as glycosidases, amidases, or endopeptidases according to their peptidoglycan cleavage site. Glycosidases cleave one of the two glycosidic bonds in the peptidoglycan chain. Amidases cleave the amide bonds between the lactyl groups of *N*-acetylmuramic acid and the L-alanine groups of the parent peptide. Endopeptidases cleave peptide bonds within the parent peptide or cross-link. Most VAPGHs are glycosidases [61].

Phage polysaccharide depolymerases are carbohydrate-active enzymes that recognize and break down bacterial envelope polysaccharides. Because they can combat biofilms, depolymerases may be effective against bacteria that form these communities, such as *Proteus mirabilis*, *E. coli*, *Streptococcus suis*, *K. pneumoniae*, and *P. aeruginosa*. Holins are small hydrophobic proteins (consisting of fewer than 150 amino acids) that regulate bacterial lysis time by guiding phage muramidases to the peptidoglycan layer. There are two types of holins: canonical and pinholins. Canonical holins form large pores in the cytoplasmic membrane through which nonspecific endolysins and other proteins are secreted into the cytoplasm. In contrast, pinholins create small holes in the membrane, leading to depolarization prior to peptidoglycan attack [61,94,95].

## 8. Synthetic AMPs

Natural AMPs present several problems, including instability, short half-lives due to sensitivity to protease degradation, potential hemolytic activity, low selectivity, cytotoxicity, and an inability to be administered orally. To address these issues and optimize their antimicrobial properties, synthetic AMPs have been developed. Inspired by natural designs and sequences, synthetic AMPs incorporate various elements through strategies such as sequence truncation, mutation, cyclization, and the introduction of unnatural amino acids and synthesizers. Synthetic AMPs can be shorter than natural AMPs—some are dipeptides—while maintaining or improving their antimicrobial properties. This ensures the presence of the minimal pharmacophore, which balances cationic charge and hydrophobicity. These compounds are more similar to small-molecule drugs and offer advantages such as proteolytic resistance and the potential for oral bioavailability. They also have significantly lower production costs than larger native AMPs. As of early 2025, APD3 contained 1299 synthetic AMPs and 231 predicted AMPs. Several small synthetic mimetics for various infectious diseases are currently in clinical development [96,97,98].

## 9. Cryptic AMPs

Several studies have shown that the proteolytic cleavage of proteins related to homeostasis leads to the generation of peptides known as cryptic host defense peptides (cHDPs), since they exhibit activities such as antimicrobial, antiviral, and immunomodulatory properties, similar to natural ones [99]. These peptides are shorter and usually have advantages such as lower susceptibility to proteolytic degradation, low toxicity, high bioavailability, lower antigenicity and lower synthesis costs. In recent years, the development of computational tools and artificial intelligence (machine learning, deep learning, and hybrid strategies) has transformed the approach for discovering new AMPs [100]. Thus, numerous cHDPs are being designed based on in silico studies of protein sequences that may include them. These analyses enable the prediction of potential AMPs based on the identification of amino acid sequences with the appropriate positive charge, hydrophobicity, and ability to form α-helix structures. They can also consider other specific features of the initial protein, such as its conservation among species of the same genus, or its other functional or structural characteristics [99,100,101].

Many studies have described the development of cHDPs with therapeutic potential. In this review, we will examine two recent cases as examples. Lata et al., [99] obtained two 15-amino-acid peptides (FKL15 and SKL15) from the ToAp2 toxin of the scorpion *Tityus obscurus*. These peptides exhibited antibacterial activity against *P. aeruginosa*, demonstrating minimal cytotoxicity towards THP-1 macrophages and BV-2 microglial cells. These authors’ results indicate that the peptides adopt a random coil structure in the presence of bacteria, acting by destroying their membranes. Saubi et al. [101] have demonstrated the presence of cAMPs in heparin-binding proteins (HBPs). All HBPs deposited in the PDB contain the CPC clip domain, including those that can also bind to lipopolysaccharides. This domain is characterized by the presence of two cationic residues and one polar residue, with conserved distances between the alpha carbons and the center of gravity of the side chain. The cAMPs identified by these authors overlap with the heparin-binding regions that contain the CPC motif. Furthermore, they observed that these cAMPs (comprising 13–26 amino acids) exhibited potent antimicrobial activity against Gram-negative bacteria. Of these proteins, HBP-5 (comprising 23 amino acids) showed the highest affinity for both heparin and lipopolysaccharides, demonstrating activity against these microorganisms in the nanomolar range. According to their results, future studies with these proteins could lead to the discovery of new antimicrobial agents.

## 10. Clinical Applications of AMPs

AMPs are active against multiple classes of bacteria, yeasts, fungi, viruses, and parasites in vitro. They are promising candidates for treating infections caused by multidrug-resistant pathogens. AMPs have several advantages, including potent antibiotic activity at low concentrations, rapid bactericidal action, and a broad spectrum of action. Additionally, they exhibit low resistance development [54,55]. However, some weaknesses must be addressed before they can be used clinically.

### 10.1. Strategies to Improve Peptide Stability

The main drawbacks hindering the clinical development of AMPs are their poor absorption, distribution, metabolism, and excretion (ADME) profiles. Digestive enzymes cleave the amide bonds in AMPs, which have poor intestinal permeability and are rapidly eliminated by the kidneys due to their high polarity and molecular weight. Systemic administration is also limited because they are susceptible to protease inactivation. Consequently, AMPs have a short half-life. Furthermore, AMPs are prone to causing hemolysis or nephrotoxicity [102].

Several strategies have been proposed to increase the metabolic stability of antimicrobial peptides (AMPs) and reduce their susceptibility to proteolytic degradation. One possibility is replacing L-amino acids with their D-enantiomeric counterparts. Mirror-image peptides and proteins comprising D-amino acids can resist proteolysis because naturally occurring proteases can only recognize and cleave proteins made of L-amino acids. For instance, the pleurocidin variant that contains only D-amino acids is impervious to degradation by trypsin, plasmin, and carboxypeptidase [103,104]. The mammalian HBcARD peptide after D-amino acid substitution also showed better stability, stronger antibacterial activity and very low hemolytic activity [48] 

Another strategy is replacing natural amino acids with α,α-disubstituted amino acids by replacing their α-hydrogen atoms with alkyl groups. These amino acids can stabilize the secondary structure of AMPs. For instance, α-aminoisobutyric acid, a non-chiral amino acid, induces helical conformation and enhances peptide stability [105].

One useful approach to enhancing peptide stability in vivo is *N*- and C-terminal modifications. Examples of these modifications include C-terminal amidation, *N*-terminal acetylation, and *N*-terminal methylation. AMPs with C-terminal amidation are prevalent in nature. Most peptides in the temporin family, for instance, are amidated at their C terminus. Additionally, side-chain modifications can make peptides resistant to proteases. Alkylation of the amine group of the lysine side chain is a useful strategy for preserving and enhancing antimicrobial potency and decreasing susceptibility to enzymatic degradation [102,103].

Intramolecular cyclization increases the formation of hydrogen bonds within peptides. This reduces solvation and enhances membrane permeability [102]. Cyclization of designed AMPs increases their activity and selectivity by reducing flexibility and enhancing stability. Several systemic AMP drugs, such as bacitracin, gramicidin S, and daptomycin, are cyclic peptides [103].

Cyclic peptides can be formed by either amide bonds (homodetic cyclization) or disulfide bonds (heterodetic cyclization). Homodetic cyclization is less common and involves intramolecular peptide bond formation. Heterodetic cyclization involves oxidizing the side-chain thiols of cysteines to create single or multiple disulfide bridges. This method is most frequently used in designing and optimizing AMPs. These bridges are more likely to form secondary or higher structures than their linear forms, decreasing susceptibility to proteolytic degradation and increasing stability. Additionally, some bacteria can produce cyclic peptides containing the amino acids lanthionine and/or methyllanthionine, which have thioether bridges. These bridges are more stable than disulfide bridges. One example of such a peptide is nisin [103].

Peptidomimetics are compounds that mimic peptide structures, but they are not composed entirely of α-amino acid residues. They are also used to improve the stability of AMPs in biological matrices. Examples of peptidomimetics include peptoids, which are poly-*N*-substituted glycines with a side chain connected to the nitrogen of the peptide backbone rather than the α-carbon; oligoacylysins, which are oligomers of acylated lysines; and β-peptides, which contain β-amino acids instead of α-amino acids. Examples of peptidomimetics include the synthetic antimicrobial peptidomimetic LTX-109, which has demonstrated in vitro bactericidal activity against several *S. aureus* isolates that are resistant to multiple classes of antimicrobial agents, and PMX-30063, a new class of defensin mimetics that is being developed to treat eye infections [102].

Teixobactin, recently isolated from uncultured bacteria, is considered a promising first-in-class drug candidate for clinical development. This natural peptide has the structural characteristics mentioned above that contribute to its stability. It is a cyclic depsipeptide that contains enduracididine (L-allo-End), an *N*-methyl-D-phenylalanine residue, and three other D-amino acid residues. Teixobactin binds to lipid II and lipid III, which results in the inhibition of peptidoglycan and teichoic acid synthesis. Teixobactin exhibits excellent activity against an array of Gram-positive pathogens, including drug-resistant strains and Mycobacterium tuberculosis [106,107].

Finally, it is worth mentioning the computational tools for AMP optimization and de novo computational methods, which have enabled the production of a large number of peptide variants with improved properties compared to the natural AMPs that inspired them [102].

In addition to the aforementioned direct modifications, other changes involving the combined use of AMP and other compounds have been employed to create synergies, improve half-life, and enhance performance. Sometimes, the combination simply consists of mixing the AMP with the other compound. Other times, it involves creating hybrid compounds or conjugates of the AMP and the other compound. This topic will be discussed in detail in Section 11 and Section 12

### 10.2. The Economic Cost of Commercial Production of AMPs

Another major challenge of bringing AMPs to market is producing them on a large scale with the required level of purity for clinical use, a process that is costly. Isolating and purifying AMPs from natural sources is labor-intensive and yields small amounts, which makes it useful only in the early stages of research. Although solid-phase peptide synthesis is highly efficient, it is also complex and expensive, costing much more than the production of conventional antibiotics. This method requires multiple chemical protection-deprotection steps for each introduced residue and uses toxic reagents in coupling or activation reactions. There is also the potential for producing peptides with sequence errors. Alternative approaches, such as genetic engineering, can be useful for obtaining AMPs with higher yields or quality. Microorganisms such as *E. coli*, *Bacillus subtilis*, *Pichia pastoris*, and *S. cerevisiae* are used in this case, and the AMPs are generally expressed as fusion proteins to facilitate microbial production and simplify purification. Several recombinant AMPs, including dermsidin, the ABP-CM4 peptide, LfcinB-W10 (derived from bovine lactoferricin), protegrin-1, the cathelicidin LL-37 peptide, and some beta-defensins, have been produced using this strategy in *E. coli*, *P. pastoris* (formerly *Komagataella phaffii*), on the other hand, is a widely used expression system for producing eukaryotic heterologous proteins. It is an ideal host because it enables numerous eukaryotic post-translational modifications, such as glycosylation, signal sequence processing, and disulfide bond formation. These modifications are necessary for cysteine-rich AMPs. This yeast has been used to produce various AMPs, including cecropins, defensins, the ABP-CM4 peptide, and the human CAP18/LL37 AMP. However, AMPs can be toxic to host cells. Therefore, using transgenic plants is also useful and offers advantages over microbial expression systems [48,108,109,110].

### 10.3. AMPs Approved for Use

The growing demand for alternative antimicrobial therapies, coupled with advancements in peptide synthesis and bioengineering techniques, makes these natural and chemically modified compounds attractive for research purposes. Although AMP-based drug discovery has increased in recent decades, the number of clinical trials and approved therapies based on these compounds remains relatively low compared to traditional antibiotics. Most of these drugs exhibit antibacterial activity, with nearly 70% being cyclic [103].

Table 2 lists the various natural and synthetic AMPs approved by the FDA or EMA (European Medicines Agency, Amsterdam, The Netherland) for clinical use [111,112,113,114,115,116].

## 11. Strategies to Improve the Properties of AMPs

### 11.1. Combinations of AMPs with Other Antimicrobials

The development of resistance to AMPs and other antibiotics renders them ineffective. One way to reduce the risk of drug resistance is to use combinations of antimicrobials that generate effective synergies. Synergistic combinations of two compounds with targets in independent pathways require two independent and simultaneous mutations to become ineffective. This approach reduces doses and side effects, improves the selectivity of the compounds, and prevents the formation of biofilms on surfaces. The latter is particularly concerning when dealing with clinical material. The use of AMPs in combination with other AMPs, other types of antibiotics, and non-direct antimicrobial cationic peptides (such as histones) has been described [117,118,119,120,121,122].

#### 11.1.1. AMP Combinations

AMPs can exhibit synergistic effects when combined with other AMPs. One example is the combination of magainins, a class of AMPs found in the skin of the African clawed frog (*Xenopus laevis*). One well-known example is the combination of magainin 2 (MAG2) and peptidyl-glycyl-leucine carboxyamide (PGLa). Both peptides act by forming transmembrane pores. When they are combined at a 1:1 ratio, membrane disruption increases by about tenfold. A membrane cross-linking study concluded that the reason this mixture is more effective is because a parallel heterodimer forms between the two peptides [118].

Synergies have also been observed when temporins are combined with other substances. Temporins are short peptides first isolated from the European red frog (*Rana temporaria*). They have a high content of leucine and isoleucine, as well as one or two positively charged amino acids. The combination of temporin B and jelleines (peptides isolated from royal jelly) exhibits a synergistic effect against *S. aureus* A170 and *L. monocytogenes*. Another example is the interaction between the human cathelicidins LL-37 and hBD-2, which effectively kill *S. aureus* through synergistic antimicrobial activity [48,118].

#### 11.1.2. Combination of AMPs with Traditional Antibiotics

The advantages of combining AMPs, cationic and cyclic lipopeptides, and antimicrobial peptide mimetics with various classes of conventional antibiotics have been documented. For example, the combination of melimine and ciprofloxacin exhibited synergistic activity against both sensitive and resistant strains of *P. aeruginosa* with fractional inhibitory concentrations (FIC) values of ≤0.5. Membrane-lytic AMPs, such as protegrin-1 or human beta-defensin-3, and intracellularly active antibiotics, such as gentamicin or rifampicin, have been shown to exhibit synergy against *MRSA*, *Micrococcus luteus*, *A. baumannii*, *K. pneumoniae*, *P. aeruginosa*, and *E. coli*, without exhibiting synergistic cytotoxic effects in normal eukaryotic cells [48,119,120,121,122,123]. Other examples include the combination of the human AMPs cathelicidin LL-37 and β-defensin 3 with the antibiotics tigecycline, moxifloxacin, piperacillin-tazobactam, and meropenem; LL-37 (residues 17–29) with chloramphenicol; nisin Z, pediocin, and colistin with penicillin or ampicillin; It is proposed that the AMPs act by altering the membrane and increasing its permeability in all of these cases. This allows the other component to access the bacterial cytoplasm and act on its intracellular targets. Several studies have also shown that AMPs enhance the antifungal activity of fluconazole, amphotericin B, clotrimazole, and other antifungal agents [118].

#### 11.1.3. Combinations of AMPs and Non-Direct Antimicrobial Cationic Peptides

Many peptides that are co-expressed and colocalized in animal tissues have been identified. While these peptides exhibit physical and structural similarities with AMPs, they lack antimicrobial activity under physiological conditions. These compounds also appear to play a crucial role in innate immunity and have been defined as non-direct antimicrobial cationic peptides (NDACPs). NDACPs primarily come from histone-derived peptides, histone variants, and the AMP family. Examples include the holohistone proteins H2A, H3, and H4, as well as their hydrolyzed fragments found in human neutrophil extracellular traps. They also include the cathelicidin and defensin families, which are present in different animals. Due to their structural characteristics, NDACPs are often misclassified as AMPs [124].

Histones and pore-forming AMPs have shown synergistic activity. For example, combining salmon histone H1 fractions with lysozyme and the AMP pleurocidin, or histones H2A and H3 with the AMPs LL-37 and magainin 2, results in a stronger antimicrobial effect than using the AMP alone. Pore-forming AMPs allow histones to enter the bacterial cytoplasm, where they inhibit global transcription and reorganize bacterial chromosomes. Furthermore, histones enhance AMP-mediated pores, promoting cytoplasmic leakage [117].

In recent years, more than 2000 NDACPs secreted by frogs have been discovered and identified. Ye et al. described the synergistic interaction between the AMP AW1 and the NDACP AW2, which are co-expressed in the frog *Amolops wuyiensis*. AW2 enhances AW1’s antibacterial activity in vitro and in vivo, decreases the development of bacterial resistance, and eliminates biofilms. The two compounds work together to damage bacterial membranes, facilitate cellular uptake, and interact with the intracellular target of AW2: bacterial genomic DNA. Additionally, they trigger the generation of reactive oxygen species (ROS), which contribute to cell death when a threshold level is reached [124].

#### 11.1.4. Antibiotic Adjuvants

The use of antibiotic adjuvants is a strategy aimed at restoring antibiotic sensitivity, broadening the antibacterial spectrum, and reducing the required dosage. These compounds act in one of two ways—directly on bacterial cells or on the host’s immune system. They can activate the immune response or facilitate the elimination of pathogens from the body. Some AMPs act as adjuvants with other antibiotics, enhancing their effectiveness. Additionally, there are examples of compounds that act as adjuvants of AMPs, which exhibit antimicrobial activity [125].

When used as adjuvants, AMPs primarily target the bacterial cell wall. This increases the wall’s permeability, allowing drugs to more easily access intracellular targets. This action is particularly crucial for Gram-negative bacteria. At other times, AMPs expressed by innate immune cells have immunomodulatory properties that influence the host’s response to infection by promoting the antimicrobial action. The concentrations at which AMPs act as adjuvants and inhibit bacterial resistance are much lower than those required for direct antimicrobial action. Recent studies in animal models confirm that AMPs function as adjuvants at nontoxic concentrations and can safely be administered in new combination chemotherapy regimens [125,126,127,128,129].

When AMP is the antimicrobial agent, it is often accompanied by another peptide that acts as an adjuvant. One example is the AMP-NDACP combination described above, in which the AMP component kills bacteria. The NDACP component, such as a histone, does not have direct antimicrobial activity; rather, it acts as an adjuvant. However, the adjuvant is not always a peptide. Certain niclosamide derivatives have been used as adjuvants to enhance the activity of polymyxin B against the *P. aeruginosa* DK2 strain isolated from patients with cystic fibrosis, as well as against other Gram-negative bacteria resistant to this antibiotic. This synergistic action has been linked to excessive ROS production, reduced ATP production, increased NADPH oxidase, and inhibition of biofilm formation [130,131].

Lytic bacteriophages infect and lyse specific bacterial cells, and they also exhibit antibiofilm activity. Combining phage and antibiotic therapies may be more effective than using either one alone. Due to their ability to target specific host organisms and replicate within bacteria, lytic bacteriophages are very useful against biofilm-mediated infections. Recently, the successful use of lytic bacteriophages in conjunction with the cyclic lipopeptide antibiotic daptomycin was reported. [132,133].

### 11.2. Covalent Conjugates with AMPs

**Figure 6:**
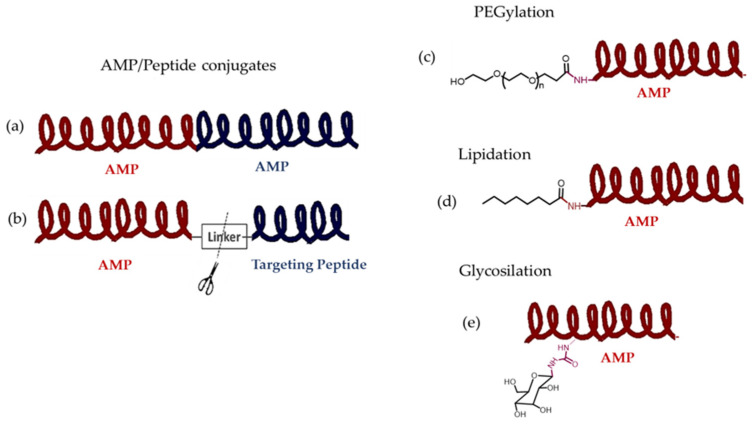
(**a**) Molecular hybrid formed from two active AMPs; (**b**) a covalent conjugate of two peptides, one of which is an AMP and the other is a targeting peptide. The peptides can bind directly or through a linker, which may or may not be cleaved. (**c**–**e**) Covalent conjugates of an active AMP with a fragment (PEG, lipid, or sugar) that lacks antimicrobial activity and acts as an adjuvant. The AMP binds to the PEG and the fatty acid via an amide bond at the *N*-terminal end. The sugar also forms an amide bond with the side chain of an asparagine (N) residue.

AMPs can be covalently linked to large molecules, such as peptides and synthetic polymers, as well as smaller molecules, including fatty acids (lipidation), sugars (glycosylation), and other antibiotics. This strategy, known as conjugation, can enhance and modulate the therapeutic efficacy of AMPs. The nature of the conjugated fragment depends on the characteristics of the AMP and its target (Figure 6) [134,135]. If the fragment conjugated to an AMP enhances its antimicrobial activity, but lacks it itself, then the fragment acts as an adjuvant to the active peptide. If both fragments are active on their own, the resulting drug is a molecular hybrid.

#### 11.2.1. Hybrid Drugs of Two AMPs and Covalently Conjugated AMP-Targeting Peptide

The hybridization of biologically active molecules is a powerful tool for designing and developing drugs to treat various diseases, including infections and cancer. Hybrid drugs, also known as “single molecule multiple targets” or “multiple ligands”, combine two or more bioactive compounds, or their pharmacophoric subunits, into a new molecular structure (Figure 6a). The structure is designed so that each compound retains its activity through direct covalent bonding or a linker. Having two or more pharmacophores in a single unit should result in greater pharmacological potency than the sum of the potencies of each individual moiety [136]. The bond joining the two units of this molecular combination can be cleavable or non-cleavable. If the bond is cleavable, the compound is a prodrug, and cleavage is desirable at the site of action. However, when the bond is non-cleavable, the hybrid drug maintains its structure throughout its entire period of residence in the organism [137].

CA-MA is a hybrid AMP resulting from the fusion of fragments of two AMPs. It contains the *N*-terminal amphipathic basic region (residues 1 to 8) of cecropin A as well as the *N*-terminal hydrophobic region (residues 1 to 12) of magainin 2. CA-MA is more effective against both Gram-negative and Gram-positive bacteria than its constituent peptides. Although CA-MA does not induce hemolysis, it retains some cytotoxicity against mammalian cells. Specific mutations in CA-MA produced a new compound, CMA3, which exhibits potent antimicrobial activity and little to no cytotoxicity toward human red blood cells and HaCaT cells, even at high concentrations. This peptide exhibits potent endotoxin-neutralizing activity against LPS in vivo and in vitro [138].

Other types of covalent conjugated peptides have been designed combining two functionally independent components linked by a short flexible linker. One component is a targeting peptide that acts as an adjuvant for the other component, a broad-spectrum AMP (Figure 6b). The targeting peptide confers selectivity to the AMP domain by binding to specific determinants on the pathogen surface, such as membrane hydrophobicity, charge, pheromone receptors, cell wall components, and characteristic virulence attributes. Kim et al. [139] described the construction of the hybrid formed by the PA2 targeting peptide, that binds specifically to the OprF porin on *P. aeruginosa*, and GNU7, a potent short AMP generated de novo. The two domains were linked by a short tripeptide (Gly3). The resulting hybrid peptide, PA2-GNU7, exhibited 16 times greater antimicrobial activity against *P. aeruginosa* than GNU7 alone, and it did not cause toxicity in the host. These same authors previously used the same strategy to develop Gram-negative-selective AMPs that can inhibit the effects of lipopolysaccharide-induced sepsis. They designed hybrid peptides by combining various rationally designed LPS-targeting peptides—including amino acids 28–34 of lactoferrin, amino acids 84–99 of the bactericidal/permeability-increasing protein, and a de novo peptide, Syn—with the potent AMP GNU7. These hybrid peptides exhibited 8- to 32-fold greater antimicrobial activity against Gram-negative bacteria, including *E. coli* and *Salmonella typhimurium*, than GNU7 [140].

#### 11.2.2. Covalent Conjugation of AMPs with Polymers

One possible solution to the problems of AMP instability and potential toxicity in mammalian cells is to obtain AMP–polymer conjugates through covalent bonding (Figure 6c). Since these conjugates enhance the antimicrobial capacity of AMPs, they can be considered adjuvants. Depending on its nature, the polymer can modulate properties such as solubility, increase protection against protease degradation, and prevent rapid renal elimination. The larger size of the conjugates decreases their cytotoxic effects on mammalian cells because they interact less with eukaryotic cell membranes. The appropriate polymer for conjugate construction is selected based on its ability to bind to the active peptide, its biocompatibility with the host, and its biodegradability. Furthermore, the conjugate must be small enough to pass through the kidneys [134,141,142]. However, conjugation can reduce or eliminate antimicrobial activity mediated by AMPs, especially when the polymer encapsulates the AMP. Therefore, it is important for these conjugates to strike a balance between the cationic and hydrophobic properties necessary for killing bacteria and the neutral and hydrophilic properties necessary for biocompatibility. High hydrophobicity increases susceptibility to α-helical folding, resulting in greater antimicrobial and hemolytic activity. Additionally, balanced amphiphilicity generally enhances antimicrobial activity and selectivity [116,134,141,143].

Hydrophilic, charge-neutral, and cationic polymers are commonly used. The first increases solubility and reduces toxicity to eukaryotic cells. However, cationic polymers also increase cytotoxicity. Polyethylene glycol (PEG) is highly soluble in water, neutral, nontoxic, and approved by the FDA for human use. The conjugation with this polymer is known as PEGylation, and can extend the half-life, improve water solubility, reduce renal clearance, prevent degradation by proteolytic enzymes, and reduce immunogenicity by shielding antigenic epitopes. This improves the overall pharmacokinetics. PEG is typically conjugated to AMP through the terminal amino group or the side chain of lysine residues. However, PEG must first be activated by forming an ester with *N*-hydroxysuccinimide [134,141].

Disadvantages of PEGylation include the difficulty of purifying and producing dispersed polymers. Furthermore, PEG induces the generation of anti-PEG antibodies, which reduces its half-life. AMPs have also been conjugated with methacrylate in dental adhesives. Spacers are inserted between the peptide and the resin to provide flexibility and stability. These conjugates exhibit bactericidal activity similar to that of the original AMPs [134].

Other polymers used are polysaccharides, such as chitosan and dextran. These polymers produce a peptidoglycan-mimetic structure that enhances interaction with bacterial membranes. Chitosan is cationic due to its amino groups and exhibits antimicrobial activity. It also conjugates with AMP through its hydroxyl and amino groups. Additionally, chitosan is biocompatible and biodegradable, making it safe for human use [134,141,142]. Various antimicrobial peptide dendrimers with biological activity against pathogens, such as the Gram-negative bacterium *P. aeruginosa*, have been conjugated to chitosan. These dendrimers are highly effective against multidrug-resistant bacteria and could be used as an alternative to antibiotics in wound dressings. The AMP dendrimer-chitosan conjugate is less toxic to mammalian cells while still exhibiting high antibacterial activity. However, the conjugate is unstable for more than 24 h [144].

Dextran is a charge-neutral polysaccharide with hydroxyl groups that provide water solubility and enable binding to AMPs. Other polymers that have been conjugated to AMPs include biodegradable polyphosphoesters, poly(ethyleneimine), poly(amidoamine), polylactides, polyacrylamides, and polyacrylates. Cui et al. [141] provide an in-depth review of these types of conjugates and the chemical methods used to prepare them. The authors analyze the relationship between the architecture of these conjugates and their antimicrobial activity. They also examine how these polymers form high-order structures, such as micelles or nanosheets, in an aqueous solution. Furthermore, they study the construction of stimuli-responsive systems. Table 3 summarizes some of their conclusions.

#### 11.2.3. Lipidation and Glycosylation of AMPs

Adding a lipid group or a sugar to an AMP can alter its hydrophobicity, secondary structure, and tendency to self-assemble without usually affecting its ability to bind to target receptors. These modifications impact the pharmacokinetics and pharmacodynamics of AMPs, enhancing their metabolic stability, membrane permeability, and bioavailability (Figure 6d,e). The presence of the glycan unit increases the hydrophilicity and bioavailability of peptides, enhancing their active transport across cell membranes. This is achieved by targeting glucose transporters on the cell membrane’s surface. The glycan unit also induces specific conformations that influence activity and increase resistance to enzymatic degradation. Consequently, the peptides’ half-life increases. [145,146].

Many articles have described the effects of these engineering devices [145,146,147,148]. One interesting example is that described by Makowska et al. [149], who analyzed the antimicrobial properties and mechanisms of action of several lipid derivatives of three natural α-helical AMPs—LL-1, LK6, and ATRA-1. They observed that the antimicrobial activity and cell selectivity were determined by the characteristics of the two components, especially the length of the lipid fragment and the charge of the peptide component. Derivatives with eight- to twelve-carbon lipid chains were the most active, but these most active analogs were also the most cytotoxic toward keratinocytes. However, they found one exception for the ATRA-1 derivatives. ATRA-1 has the highest net positive charge of the three peptides, and this charge is probably the factor that determines cell selectivity.

In a similar way, Tortorella et al. [150] constructed the glycopeptide G-LL-III through the *N*-glycosylation of an asparagine residue in AMP LL-III. This new system offered greater resistance to proteolytic enzyme activity without modification of the action mechanism. The glycopeptide can adopt a helical conformation when it binds to a lipid bilayer, forming lipid domains that allow access to the cytoplasm of a cell.

## 12. Nanostructures

Liposomes, the first nanostructures used for drug delivery, were developed at the end of the 20th century. Since then, extensive research has been conducted on these systems, resulting in the development of many similar devices. Nanostructures are encapsulation systems that transport and release substances, particularly active ingredients, in a controlled manner. Nanodelivery systems loaded with AMPs safely package the peptides before they exert their antimicrobial actions. The instability of peptides in biological media and their low gastrointestinal absorption make developing orally administered pharmaceutical forms difficult. These nanostructures can modulate the peptides’ pharmacokinetic and pharmacodynamic properties, protecting them from protease-mediated biodegradation and increasing their chemical stability. These new delivery systems and formulations demonstrate greater activity than single AMPs, overcoming the barriers to their clinical development. They facilitate targeted delivery to sites of infection and control the release rate of the peptides, thereby reducing toxic side effects. Furthermore, these systems could enhance the antibacterial effect by acting synergistically with AMPs [109,151].

**Figure 7:**
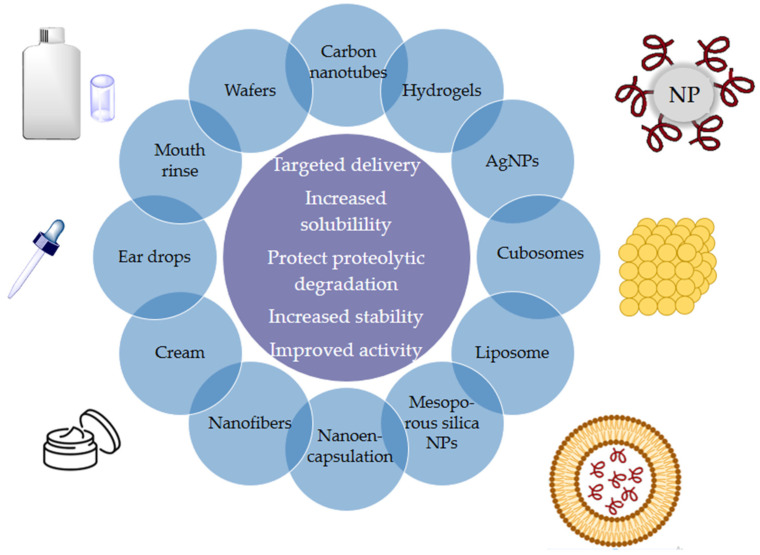
Different formulations of AMPs for improving their antimicrobial action by particular mechanisms. From the information described in Asif et al. [112].

Useful materials for producing AMP nanocarriers include metal nanoparticles, vesicles, microgels, hydrogels, nanofibers, carbon nanotubes and mesoporous silica nanoparticles among others (see Figure 7) [134,152,153,154]. Nanocarriers transport drugs via physical encapsulation or adsorption, releasing them into target cells through contact, adsorption, or endocytosis. These devices can overcome challenges such as inefficient drug absorption and the inability to cross biological membranes. Typically, the drug and the nanocarrier are linked by a stable or cleavable covalent bond. In the latter case, the drug is released from the conjugate when it reaches its intracellular target. Intracellular cleavage is caused by enzymes present at the release site or by endogenous and exogenous stimuli, such as the microenvironment of an infection, the reducing properties of cytosolic glutathione, or the acidic environment of endosomes formed during endocytosis. The biodegradability of the nanocarrier influences its efficacy, safety, and applicability in medical treatments [153,155,156,157]. As discussed in the previous section, conjugated AMP–polymers can form higher-order structures, such as micelles or nanosheets (see Table 3) [141].

Asif et al.’s review [112] provides a thorough description of the different systems used for this purpose (Figure 7). In this revision, we will only show a few illustrative examples.

Microgels and hydrogels are classes of biomaterials consisting of cross-linked, hydrated polymers that can exhibit tunable swelling responses to a wide range of parameters. These microgels can also be designed for remote triggering by light or magnetic field. Responsiveness can also be reached to disease-specific stimuli, e.g., for glucose-triggered insulin release, or for antibiotics release triggered by bacterial proteases. Because of their properties, hydrogels can be customized and used in a variety of ways. This makes them ideal for incorporating or tethering a wide range of antimicrobial agents [154,158,159].

Due to their ability to mimic the composition and physiochemical properties of the human extracellular matrix, hydrogels have a high potential for use as wound dressings. They are highly biocompatible and biodegradable in vivo. However, hydrogels alone are mechanically weak, adhere poorly to wet tissues, and exhibit limited antimicrobial activity. Several photoinduced hydrogels have been used to embed AMPs. This system exhibits the desired degradation behavior, swelling kinetics, and rheological characteristics of an ideal wound dressing, promoting faster healing [154].

Self-assembling hydrogels can also be entirely peptide-based and form three-dimensional fibrous networks via non-covalent cross-linking between peptides through ionic, hydrophobic, hydrogen, or π-π stacking interactions. AMP self-forming hydrogels can include additional networks that increase their functionality. For example, they can integrate hyaluronic acid to accelerate wound healing or adenosine diphosphate (ADP), which is important for platelet aggregation and coagulation. Additionally, systems based on temperature, pH, and enzymatic cleavage of hydrogel-bound AMP are being studied to control AMP delivery with these carriers. Because high concentrations of AMP can be cytotoxic, it would be desirable to develop hydrogels with minimal AMP concentrations that maximize bactericidal properties while minimizing unwanted cytotoxicity. One way to achieve this would be to explore the use of multiple AMPs with synergistic effects, as described above [154].

Polyelectrolytes have properties similar to those of polymers and electrolytes, and they can be used as delivery systems for AMPs. As Borro et al. describe in their review [158], polyelectrolytes and hydrogels have many similarities. Both types of systems have dense structures that are primarily affected by osmotic deswelling and have similar factors that determine AMP loading and release. AMPs with a large net positive charge can form strong complexes by simply mixing with non-crosslinked polyelectrolytes. Unlike traditional microgels, the “cross-links” in this case form through physical association rather than covalent coupling. Furthermore, covalently cross-linked microgels are often loaded with peptides after synthesis and purification. In contrast, peptide loading and particle formation occur simultaneously in a one-step process in polyelectrolyte complexation.

Metallic nanoparticles (NPs) are extremely important in medical science due to their unique characteristics, such as shape, charge, size, high surface-to-mass ratio, and high reactivity. NPs have been shown to exhibit antimicrobial activity against both Gram-negative and Gram-positive bacteria and have been proposed as an alternative to conventional antibiotics [160]. Different mechanisms of action have been described depending on the material, shape, and size of their core. Their nanometric size enables them to interact directly with cell membranes and penetrate them, thereby altering their form and function. Furthermore, they release metal ions that generate ROS internally, which attack proteins and affect enzymatic activity. These metal ions can also cause structural changes in cells and interact with phosphorus-containing compounds, such as DNA. This prevents unwinding, replication, and transcription, ultimately leading to cell death. Due to their antimicrobial properties, silver and gold nanoparticles are of particular interest and have been used to treat infections caused by antibiotic-resistant strains such as *S. aureus*, *E. faecium*, *E. faecalis*, *E. coli*, *V. cholerae*, *S. typhimurium*, and *Salmonella dysenteriae* [54,160].

Many studies have demonstrated the effectiveness of gold (AuNPs) and silver (AgNPs) nanoparticles as AMP nanocarriers. AMPs can bind directly to the nanoparticles via uncontrolled interactions, such as van der Waals forces or metal-sulfur bonds, or indirectly via a bifunctional linker that binds to both the peptide and the functionalized NP [161]. Coating the surface of AgNPs with peptides or proteins can increase their colloidal stability and bioavailability while reducing immunogenicity. Binding AMPs to the surface of NPs is also a suggested method for improving their stability under physiological conditions. Several studies have compared the antibacterial properties of AMP (or protein) conjugates with AgNPs to the activity of each component separately. For example, conjugating AMPs, such as protegrin-1, indolicidin, protamine, histones, and lysozyme, with AgNPs maintains synergy and increases antimicrobial activity compared to unconjugated NPs or AMPs. Regardless of the hemolytic activity of free polypeptides, all conjugates exhibited low hemolytic activity, demonstrating that this strategy enhances antimicrobial power while reducing the toxicity of membranolytic AMPs [162]. Another study showed that conjugating daptomycin with silver nanoclusters increased its bactericidal activity compared to using daptomycin or silver nanoclusters alone [163].

Gao et al. synthesized AgNPs protected by a 13-amino acid peptide with two functional regions: one for antibacterial activity and the other for reducing and stabilizing the AgNPs with cysteine residues at their C-terminus. The researchers observed that these conjugates exhibited more favorable antimicrobial activity against four bacterial strains than the peptide or AgNPs alone. After peptide protection, the cytotoxicity of the AgNPs was dramatically reduced [164].

Recently, the preparation and characterization of an AMP@PDA@AgNPs nanocomposite were described. This material consists of AMP and polydopamine (PDA), a polymer formed by the oxidative polymerization of dopamine. PDA exhibits excellent adhesion and cytocompatibility properties. Compared to AgNPs or AMP alone, the nanocomposite exhibited superior biocompatibility and antimicrobial activity against both Gram-negative and Gram-positive bacteria. Furthermore, the nanocomposite effectively destroyed bacterial biofilms by inhibiting the expression of related genes [165].

Cubosomes are a type of stable, nanostructured material with the unique ability to encapsulate and protect peptide drugs from degradation. Consisting of folded lipid bilayers curved in three-dimensional space, they have interwoven water channels. Their amphipathic structure enables the incorporation of hydrophilic drugs into the aqueous channels, hydrophobic drugs into the lipid bilayers, and amphiphilic drugs into the bilayer-water interface. Compared to conventional liposomes, cubosomes have a larger membrane surface area for drug loading. Several AMPs have been successfully incorporated into cubosomes. For instance, cubosome systems have been employed to deliver the AMP LL-37 topically to treat skin infections caused by *S. aureus*, which demonstrates that LL-37 is shielded from proteolytic degradation. [166].

## 13. Conclusions

From the 1940s to the 1960s, many antibiotic families with different structures were discovered and developed for clinical use. This solved the major problem of bacterial infections, which had claimed many lives up to that time. However, the use and overuse of these new drugs led to a new problem—the development of resistance to these compounds. This posed a new challenge—the need to discover new molecules and safe strategies for this purpose. In recent years, few compounds have been approved, most of which are molecules or combinations of molecules belonging to the families traditionally used, with slight structural modifications being introduced [167]. Therefore, it is necessary to discover new entities with different characteristics from those already used. In this regard, endogenous peptides known as AMPs have recently attracted the interest of many researchers. These relatively small compounds have antimicrobial activity. They are molecules produced naturally by the organism as a defense mechanism against pathogen invasion. The structure, mode of action, and potential activity of many of these have been determined. However, the effectiveness of these compounds is limited by pharmacokinetic issues because they are large, charged molecules that are sensitive to proteases and acidic environments. Currently, research focuses on solving these problems in different ways, such as combining AMPs with each other or with other compounds that generate synergies, modifying their structure by binding them to polymers, lipids, or sugars, and using different types of nanostructured materials, such as metallic nanoparticles, hydrogels, cubosomes, and nanotubes. The use of nanostructured systems carrying drugs has a promising future since they allow for the control of parameters such as nonspecific toxicity, pharmacokinetics, and pharmacodynamics. These materials determine and influence fundamental aspects of drug transit through the body, including dosage, duration of action, and location of effect—factors that determine the drug’s efficacy.

In this work, we reviewed the characteristics of AMPs, the known classes, and the strategies proposed to solve the practical problems involved in their use and clinical implementation. It is important to note that this cannot be the only strategy to combat antibiotic resistance; it must be combined with others, such as identifying new natural products with antimicrobial properties, developing useful antibodies, and gene therapy.

## Figures and Tables

**Table 1:** Examples of different mechanisms of antibiotic resistance ^1^.

Mechanism	Example
Modification of the pharmacological target	Modification of the structure of penicillin-binding proteins, which allow for the synthesis of peptidoglycan, the component that makes up the cell wall (in MRSA)
	Modification of the last amino acid of peptidoglycan (in glycopeptide-resistant bacteria)
	Remodeling of the chemical structure of bacterial gyrase and/or topoisomerase IV (in fluoroquinolone-resistant bacteria)
	Expression of *vanA* and *vanB* genes products, which modify cell wall precursors and decrease vancomycin binding (in glycopeptide-resistant bacteria)
	Expression of *ermb* gene, which codifies ribosome methylase enzyme. This enzyme modifies rRNA and impedes drug binding to the ribosome (in macrolide and tetracycline-resistant bacteria)
Alterations in membrane permeability that prevent antibiotic penetration into the cell	Decreased sensitivity of porins to beta-lactam antibiotics and fluoroquinolones.
Active pumping of the antibiotic out of the cell	*MSF* provides resistance to fluoroquinolones, macrolides, linezolid, … (in strains of *S. aureus* and *Escherichia coli*)
	*MATE* provides resistance to fluoroquinolones and some aminoglucosides (in *Neisseria gonorrhoeae* and *S. aureus*)
Enzymatic inactivation of the antibiotic	Modification of macrolides by esterases that hydrolyze their lactone ring (in *Salmonella enterica*, *Pseudomonas* spp., *Vibrio cholera*, and *Klebsiella* spp.)
	Phosphorylation, adenylation or acetylation of aminoglycosides (in kanamycin, neomycin, and paromomycin-resistant bacteria)

^1^ Information obtained from [6,7].

**Table 2:** FDA/EMA approved AMPs.

Compound	Via	Application	Target Species	Year of Approval
Anidulafungin (semisynthetic lipopeptide)	Intravenous	Treatment of invasive candidiasis	Fungi (mainly *Candida*)	2006
^1^ Atazanavir (azapeptide)	Oral	HIV-infection	HIV-1	2014
^1^ Bacitracin (Cyclic peptide)	Topical	Pneumonia, localized skin and eye infections and wound infections	Gram-positive	1948
^1^ Boceprevir	Oral	Chronic hepatitis C genotype 1	Hepatitis C virus	2011
^1^ Bulevirtide	Subcutaneous	Chronic hepatitis D	Hepatitis D virus	2015 (EMA)
^3^ Carfilzomib	Intravenous	Multiple myeloma cells		2012
Caspofungin (semisynthetic lipopetide)	Intravenous	Fungal infections	*Candida* and *Aspergillus* spp. and other fungi	2001
^2^ Colistin (lipopeptide)	Auricular (otic)	Infections caused by Gram-negative bacteria resistant to other antibiotics	*P. aeruginosa* and some Gram-negative bacilli	1959
Dalbavancin (semisynthetic lipoglycopeptide)	Intravenous	Acute bacterial skin infections	*S. aureus* *S. pyogenes* *Streptococcus agalactiae*	2014
^2^ Daptomycin (lipopeptide)	Intravenous	Particularly complicated infections of the skin and its structures	*S.aureus*	2003
^1^ Enfuvirtide	Subcutaneous	Infections produced by HIV-1	HIV-1	2003
^1^ Glecaprevir	Oral	Chronic hepatitis C genotype 1–6	Hepatitis C virus	2017
^2^ Gramicidin D	Topical	Infected surface wounds, as well as eye, nose, and throat infections	Most Gram-positive and some Gram-negative bacteria	1955
^1^ Invinavir	Oral	Treatment of HIV infection	HIV-1	1996
^3^ Liraglutide	Subcutaneous	Improve blood sugar, reduce the risk of major cardiovascular events		2010
^1^ Lopinavir	Oral	HIV infections	HIV-1	2000
^2^ Interferon alfa 2B (leucocyte fraction of human blood)	Oral	Hepatitis B infections and cancer	Hepatitis B virus	1986
Micafungin (Semisynthetic lipopeptide)	Intravenous		Fungi (main *Candida*)	2005
^1^ Nelfinavir	Oral	HIV infections	HIV-1	1997
^2^ Nisin (polycyclic peptide)		Food preservative	Gram-positive bacteria and their spores	1988
Oritavancin (Semisynthetic glycopeptide)	Intravenous	Skin and skin structure infections	*S. aureus*	2014
Rezafungin (Semisynthetic lipopeptide)	Intravenous	Fungal infections	*Candida*, *Aspergillus*, and *Pneumocystis* spp.	2023
^1^ Ritonavir (Biomimetic peptide)	Oral	HIV infection	HIV-1	2015
^3^ Romidepsin (naturally occurring bicyclic depsipeptide)	Intravenous	Cutaneous T-cell lymphoma		2009
^1^ Saquinavir	Oral	HIV infection	HIV-1	1995
Teicoplanin (Semisynthetic glycopeptide)	Intravenous	Life threatening Gram-positive bacterial infections	*E. faecalis* and MRSA	1988 (EMA)
Telaprevir (Semisynthetic linear peptide)	Oral	Chronic hepatitis C	Hepatitis C virus	2011
Telavancin (Semisynthetic lipoglycopeptide, derivative of vancomycin)	Intravenous	Complicated infections of skin and skin structures	*S. aureus*, *S. pyogenes*, and vancomycin-susceptible *E. faecalis*	2009
^1^ Tesamorelin	Subcutaneous	Reduce the amount of abdominal fat that is excessive and keep it from returning	HIV-1	2010
^2^ Vancomycin (glycopeptide)	Intravenous, oral	MRSA infections	MRSA	1954

^1^ Synthetic peptides, ^2^ Natural peptides, ^3^ Not used for infections.

**Table 3:** Properties of AMP–polymer conjugates (based on [141]).

**Polymer Architecture: Properties of the AMP–Polymer Conjugates** **and Antimicrobial Performance**	
^1^ Linear AMP— polymer conjugates 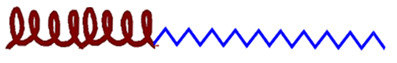	A single AMP is attached to a linear polymer chain. Improved stability and biocompatibility. The antimicrobial activity and cytotoxicity depend on the size of the polymer: Conjugation to polymers decreases a peptide’s cytotoxic effects and antibacterial activity because the peptide is likely buried within the polymer. Generally, shortening the polymer increases its antimicrobial effect.
^1^ Comb/brush AMP— polymer conjugates 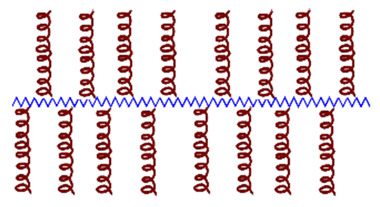	Macromolecular (polymeric or peptide) pendent groups on a main chain, which can be a polymer or a peptide. The local concentration of AMPs in a single polymer molecule generates potent bactericidal conjugates. The bactericidal activity and cytotoxic effects of these conjugates can be modulated by modifying the density, the length and the orientation of the AMPs and the length of the polymer chain. In general, conjugating AMPs to this scaffold reduces cytotoxicity while preserving or enhancing their bactericidal activity.
^1^ Star-shaped AMP— polymer conjugates 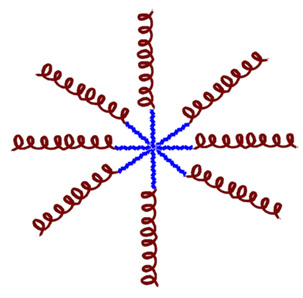	Multiple polymer chains (arms) emanating from a central core. The balance of bactericidal activity, mammalian cell toxicity, protease resistance, and conjugate aggregation is influenced by the number and length of the arms, as well as their composition. They exhibit a high local concentration of AMP and potent antimicrobial activity.
^1^ Hyperbranched AMP– polymer conjugates 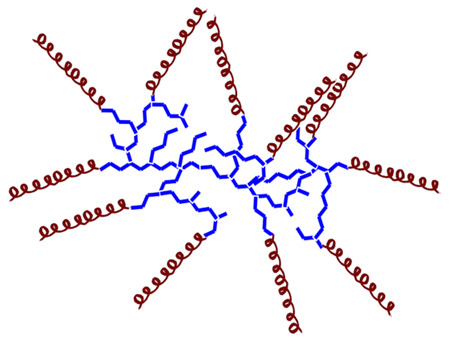	There are not many studies of conjugates with this architecture. Generally, they exhibit reduced antimicrobial activity compared to AMP alone. However, they also demonstrate lower toxicity to mammalian cells and improved compatibility with blood components. These properties can be modulated by hyperbranched architecture, composition, degree of branching, molecular weight, and terminal group functionality. Decreasing the molecular weight of the hyperbranched polymer is related to enhanced antimicrobial activity.
**Supramolecular Assembly of AMP–Polymer Conjugates**	
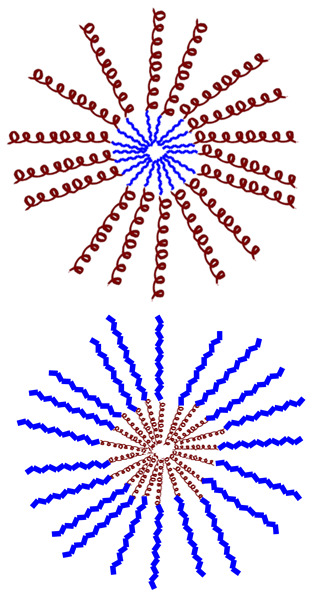	^1^ Micelles formed by the assembly of a hydrophilic AMP attached to a hydrophobic polymer. Increased antimicrobial activity. Reduced toxicity. ^1^ Micelles formed by the assembly of a hydrophobic AMP attached to a hydrophilic polymer. They exhibit lower cytotoxicity and protection against degradation.

^1^ Red color: AMP; Blue color: Polymer.

## Data Availability

No new data were created or analyzed in this study.

## Supplementary Materials

The following supporting information can be downloaded at: https://www.mdpi.com/article/10.3390/molecules30153070/s1, Table S1: AMPs of animal origin; Table S2: Bacterial ribosomal AMPs; Table S3: Microbial Nonribosomal AMPs.

## Abbreviations

The following abbreviations are used in this manuscript:

aa	Amino acid(s)
ABPs	Antibacterial peptides
Aib	α-aminoisobutyric acid
AMPs	Antimicrobial peptides
APD	Antimicrobial peptide database
cHDPs	Cryptic host defense peptides
EMA	European medicines agency
FDA	United States food and drug administration
FIC	Fractional inhibitory concentrations
HBPs	Heparin-binding proteins
HDPs	Host defense molecules
LAB	Lactic acid bacteria
LPS	Lipopolysaccharides
MATE	Multidrug and toxic compound extrusion family
MSF	Major facilitator superfamily
MRSA	Methicillin-resistant *Staphylococcus aureus*
NDACPs	Non-direct antimicrobial cationic peptides
NP	Nanoparticle
NRPs	Non-ribosomal peptides
NRPSs	Non-ribosomal peptide synthetases
PDB	Protein Data Bank
PrAMPs	Proline-rich AMPs
PTM	Post-translational modification
VAPGHs	Virion-associated peptidoglycan hydrolases
VRE	Vancomycin-resistant *Enterococci*
WHO	World Health Organization

## References

[1] Bush K., Bradford P.A. (2016). β-Lactams and β-Lactamase Inhibitors: An Overview. Cold Spring Harb. Perspect. Med..

[2] Spagnolo F., Trujillo M., Dennehy J.J. (2021). Why Do Antibiotics Exist?. mBio.

[3] Haq S.U., Ling W., Aqib A.I., Danmei H., Aleem M.T., Fatima M., Ahmad S., Gao F. (2025). Exploring the Intricacies of Antimicrobial Resistance: Understanding Mechanisms, Overcoming Challenges, and Pioneering Innovative Solutions. Eur. J. Pharmacol..

[4] Davies J., Davies D. (2010). Origins and Evolution of Antibiotic Resistance. Microbiol. Mol. Biol. Rev..

[5] Burki T.K. (2021). Development of New Antibacterial Agents: A Sense of Urgency Needed. Lancet Respir. Med..

[6] Baran A., Kwiatkowska A., Potocki L. (2023). Antibiotics and Bacterial Resistance—A Short Story of an Endless Arms Race. Int. J. Mol. Sci..

[7] Martinez J.L. (2014). General Principles of Antibiotic Resistance in Bacteria. Drug Discov. Today Technol..

[8] Gillings M.R., Paulsen I.T., Tetu S.G. (2017). Genomics and the Evolution of Antibiotic Resistance. Ann. N. Y. Acad. Sci..

[9] Ho C.S., Wong C.T.H., Aung T.T., Lakshminarayanan R., Mehta J.S., Rauz S., McNally A., Kintses B., Peacock S.J., De La Fuente-Nunez C. (2025). Antimicrobial Resistance: A Concise Update. Lancet Microbe.

[10] Sánchez-López E., Gomes D., Esteruelas G., Bonilla L., Lopez-Machado A.L., Galindo R., Cano A., Espina M., Ettcheto M., Camins A. (2020). Metal-Based Nanoparticles as Antimicrobial Agents: An Overview. Nanomaterials.

[11] Mondal S.K., Chakraborty S., Manna S., Mandal S.M. (2024). Antimicrobial Nanoparticles: Current Landscape and Future Challenges. RSC Pharm..

[12] Luo L., Huang W., Zhang J., Yu Y., Sun T. (2024). Metal-Based Nanoparticles as Antimicrobial Agents: A Review. ACS Appl. Nano Mater..

[13] Uddin Mahamud A.S., Nahar S., Ashrafudoulla M., Park S.H., Ha S.D. (2024). Insights into Antibiofilm Mechanisms of Phytochemicals: Prospects in the Food Industry. Crit. Rev. Food Sci. Nutr..

[14] Murugan S., Senthilvelan T., Govindasamy M., Thangavel K. (2025). A Comprehensive Review on Exploring the Potential of Phytochemicals and Biogenic Nanoparticles for the Treatment of Antimicrobial-Resistant Pathogenic Bacteria. Curr. Microbiol..

[15] Tyagi J.L., Gupta P., Ghate M.M., Kumar D., Poluri K.M. (2024). Assessing the Synergistic Potential of Bacteriophage Endolysins and Antimicrobial Peptides for Eradicating Bacterial Biofilms. Arch. Microbiol..

[16] Chavan R., Purandare K. (2025). Bacteriophage Therapy Inspired New Age Technologies to Control Antimicrobial Resistance. J. Umm Al-Qura Univ. Appll. Sci..

[17] Kuchay R.A.H. (2024). Novel and Emerging Therapeutics for Antimicrobial Resistance: A Brief Review. Drug Discov. Ther..

[18] Berger I., Loewy Z.G. (2024). Antimicrobial Resistance and Novel Alternative Approaches to Conventional Antibiotics. Bacteria.

[19] La Guidara C., Adamo R., Sala C., Micoli F. (2024). Vaccines and Monoclonal Antibodies as Alternative Strategies to Antibiotics to Fight Antimicrobial Resistance. Int. J. Mol. Sci..

[20] Moretta A., Scieuzo C., Petrone A.M., Salvia R., Manniello M.D., Franco A., Lucchetti D., Vassallo A., Vogel H., Sgambato A. (2021). Antimicrobial Peptides: A New Hope in Biomedical and Pharmaceutical Fields. Front. Cell. Infect. Microbiol..

[21] Wang G., Li X., Wang Z. (2016). APD3: The Antimicrobial Peptide Database as a Tool for Research and Education. Nucleic Acids Res..

[22] Reddy K.V.R., Yedery R.D., Aranha C. (2004). Antimicrobial Peptides: Premises and Promises. Int. J. Antimicrob. Agents.

[23] Lehrer R.I., Ganz T. (1999). Antimicrobial Peptides in Mammalian and Insect Host Defence. Curr. Opin. Immunol..

[24] Liang W., Diana J. (2020). The Dual Role of Antimicrobial Peptides in Autoimmunity. Front. Immunol..

[25] Hassan M., Kjos M., Nes I.F., Diep D.B., Lotfipour F. (2012). Natural Antimicrobial Peptides from Bacteria: Characteristics and Potential Applications to Fight Against Antibiotic Resistance. J. Appl. Microbiol..

[26] Arbulu S., Kjos M. (2024). Revisiting the Multifaceted Roles of Bacteriocins: The Multifaceted Roles of Bacteriocins. Microb. Ecol..

[27] Anurag Anand A., Amod A., Anwar S., Sahoo A.K., Sethi G., Samanta S.K. (2024). A Comprehensive Guide on Screening and Selection of a Suitable AMP Against Biofilm-Forming Bacteria. Crit. Rev. Microbiol..

[28] Batoni G., Maisetta G., Esin S. (2016). Antimicrobial Peptides and Their Interaction with Biofilms of Medically Relevant Bacteria. Biochim. Biophys. Acta (BBA) Biomembr..

[29] Haney E.F., Straus S.K., Hancock R.E.W. (2019). Reassessing the Host Defense Peptide Landscape. Front. Chem..

[30] Luong H.X., Ngan H.D., Thi Phuong H.B., Quoc T.N., Tung T.T. (2022). Multiple Roles of Ribosomal Antimicrobial Peptides in Tackling Global Antimicrobial Resistance. R. Soc. Open Sci..

[31] Noor H.B., Mou N.A., Salem L., Shimul M.F.A., Biswas S., Akther R., Khan S., Raihan S., Mohib M.M., Sagor M.A.T. (2020). Anti-Inflammatory Property of AMP-Activated Protein Kinase. Anti-Inflamm. Anti-Allergy Agents Med. Chem..

[32] Tornesello A.L., Borrelli A., Buonaguro L., Buonaguro F.M., Tornesello M.L. (2020). Antimicrobial Peptides as Anticancer Agents: Functional Properties and Biological Activities. Molecules.

[33] Wang G. (2022). Unifying the Classification of Antimicrobial Peptides in the Antimicrobial Peptide Database. Methods in Enzymology.

[34] Huan Y., Kong Q., Mou H., Yi H. (2020). Antimicrobial Peptides: Classification, Design, Application and Research Progress in Multiple Fields. Front. Microbiol..

[35] Straus S.K. (2024). Tryptophan- and Arginine-Rich Antimicrobial Peptides: Anti-Infectives with Great Potential. Biochim. Biophys. Acta (BBA) Biomembr..

[36] Dzurová L., Holásková E., Pospíšilová H., Schneider Rauber G., Frébortová J. (2024). Cathelicidins: Opportunities and Challenges in Skin Therapeutics and Clinical Translation. Antibiotics.

[37] Stączek S., Kunat-Budzyńska M., Cytryńska M., Zdybicka-Barabas A. (2024). Proline-Rich Antimicrobial Peptides from Invertebrates. Molecules.

[38] Pirtskhalava M., Vishnepolsky B., Grigolava M., Managadze G. (2021). Physicochemical Features and Peculiarities of Interaction of AMP with the Membrane. Pharmaceuticals.

[39] Díaz M.D., Palomino-Schätzlein M., Corzana F., Andreu C., Carbajo R.J., Del Olmo M., Canales-Mayordomo A., Pineda-Lucena A., Asensio G., Jiménez-Barbero J. (2010). Antimicrobial Peptides and Their Superior Fluorinated Analogues: Structure–Activity Relationships as Revealed by NMR Spectroscopy and MD Calculations. ChemBioChem.

[40] Sudheendra U.S., Dhople V., Datta A., Kar R.K., Shelburne C.E., Bhunia A., Ramamoorthy A. (2015). Membrane Disruptive Antimicrobial Activities of Human β-Defensin-3 Analogs. Eur. J. Med. Chem..

[41] Berman H., Henrick K., Nakamura H. (2003). Announcing the Worldwide Protein Data Bank. Nat. Struct. Mol. Biol..

[42] Wang G., Wang G. (2017). Discovery, classification and functional diversity of antimicrobial peptides. Antimicrobial Peptides: Discovery, Design and Novel Therapeutic Strategies.

[43] Bourbigot S., Dodd E., Horwood C., Booth V. (2008). NMR Structure of the Antimicrobial Peptide RP-1 Bound to SDS Micelles: 2rlg. Biopolymers.

[44] Shenkarev Z.O., Balandin S.V., Trunov K.I., Paramonov A.S., Sukhanov S.V., Barsukov L.I., Arseniev A.S., Ovchinnikova T.V. (2011). Molecular Mechanism of Action of β-Hairpin Antimicrobial Peptide Arenicin: Oligomeric Structure in Dodecylphosphocholine Micelles and Pore Formation in Planar Lipid Bilayers. Biochemistry.

[45] Reiser J.B., Teyton L., Wilson I.A. (2004). Crystal Structure of the *Drosophila* Peptidoglycan Recognition Protein (PGRP)-SA at 1.56Å Resolution. J. Mol. Biol..

[46] Stewart J., Shawon J., Ali M.A., Williams B., Shahinuzzaman A.D.A., Rupa S.A., Al-Adhami T., Jia R., Bourque C., Faddis R. (2024). Antiviral Peptides Inhibiting the Main Protease of SARS-CoV-2 Investigated by Computational Screening and In Vitro Protease Assay. J. Pept. Sci..

[47] Erdem Büyükkiraz M., Kesmen Z. (2022). Antimicrobial Peptides: Mechanism of Action, Activity and Clinical Potential. J. Appl. Microbiol..

[48] Zhang Q.-Y., Yan Z.-B., Meng Y.-M., Hong X.-Y., Shao G., Ma J.-J., Cheng X.-R., Liu J., Kang J., Fu C.-Y. (2021). Antimicrobial Peptides: Mechanism of Action, Activity and Clinical Potential. Mil. Med. Res..

[49] Seyfi R., Kahaki F.A., Ebrahimi T., Montazersaheb S., Eyvazi S., Babaeipour V., Tarhriz V. (2020). Antimicrobial Peptides (AMPs): Roles, Functions and Mechanism of Action. Int. J. Pept. Res. Ther..

[50] Nayab S., Aslam M.A., Rahman S.U., Sindhu Z.U., Sajid S., Zafar N., Razaq M., Kanwar R. (2022). Amanullah A Review of Antimicrobial Peptides: Its Function, Mode of Action and Therapeutic Potential. Int. J. Pept. Res. Ther..

[51] Francis F., Chaudhary N. (2023). Antimicrobial Peptides: Features and Modes of Action. Antimicrobial Peptides.

[52] Zheng S., Tu Y., Li B., Qu G., Li A., Peng X., Li S., Shao C. (2025). Antimicrobial Peptide Biological Activity, Delivery Systems and Clinical Translation Status and Challenges. J. Transl. Med..

[53] Gagandeep K.R., Narasingappa R.B., Vyas G.V. (2024). Unveiling Mechanisms of Antimicrobial Peptide: Actions beyond the Membranes Disruption. Heliyon.

[54] León-Buitimea A., Garza-Cárdenas C.R., Garza-Cervantes J.A., Lerma-Escalera J.A., Morones-Ramírez J.R. (2020). The Demand for New Antibiotics: Antimicrobial Peptides, Nanoparticles, and Combinatorial Therapies as Future Strategies in Antibacterial Agent Design. Front. Microbiol..

[55] Fatima H., Goel N., Sinha R., Khare S.K. (2021). Recent Strategies for Inhibiting Multidrug-Resistant and β-Lactamase Producing Bacteria: A Review. Colloids Surf. B Biointerfaces.

[56] Kim J., Cho B.-H., Jang Y.-S. (2023). Understanding the Roles of Host Defense Peptides in Immune Modulation: From Antimicrobial Action to Potential as Adjuvants. J. Microbiol. Biotechnol..

[57] Xu D., Lu W. (2020). Defensins: A Double-Edged Sword in Host Immunity. Front. Immunol..

[58] Patterson-Delafield J., Martinez R.J., Lehrer R.I. (1980). Microbicidal Cationic Proteins in Rabbit Alveolar Macrophages: A Potential Host Defense Mechanism. Infect. Immun..

[59] Solanki S.S., Singh P., Kashyap P., Sansi M.S., Ali S.A. (2021). Promising Role of Defensins Peptides as Therapeutics to Combat Against Viral Infection. Microb. Pathog..

[60] Kumaresan V., Kamaraj Y., Subramaniyan S., Punamalai G. (2024). Understanding the Dynamics of Human Defensin Antimicrobial Peptides: Pathogen Resistance and Commensal Induction. Appl. Biochem. Biotechnol..

[61] Bin Hafeez A., Jiang X., Bergen P.J., Zhu Y. (2021). Antimicrobial Peptides: An Update on Classifications and Databases. Int. J. Mol. Sci..

[62] Salnikov E., Adélaïde M., Ramos-Martín F., Saad A., Schauer J., Cremanns M., Rima M., Aisenbrey C., Oueslati S., Naas T. (2025). Cathelicidin-BF: A Potent Antimicrobial Peptide Leveraging Charge and Phospholipid Recruitment Against Multidrug-Resistant Clinical Bacterial Isolates. J. Am. Chem. Soc..

[63] Talapko J., Meštrović T., Juzbašić M., Tomas M., Erić S., Horvat Aleksijević L., Bekić S., Schwarz D., Matić S., Neuberg M. (2022). Antimicrobial Peptides—Mechanisms of Action, Antimicrobial Effects and Clinical Applications. Antibiotics.

[64] Pan L., Zhang X., Gao Q. (2021). Effects and Mechanisms of Histatins as Novel Skin Wound-Healing Agents. J. Tissue Viability.

[65] Fazly Bazzaz B.S., Seyedi S., Hoseini Goki N., Khameneh B. (2021). Human Antimicrobial Peptides: Spectrum, Mode of Action and Resistance Mechanisms. Int. J. Pept. Res. Ther..

[66] Luong A.D., Buzid A., Luong J.H.T. (2022). Important Roles and Potential Uses of Natural and Synthetic Antimicrobial Peptides (AMPs) in Oral Diseases: Cavity, Periodontal Disease, and Thrush. J. Funct. Biomater..

[67] Conlon J.M., Mechkarska M. (2014). Host-Defense Peptides with Therapeutic Potential from Skin Secretions of Frogs from the Family Pipidae. Pharmaceuticals.

[68] Mohamed Abd El-Aziz T., Soares A.G., Stockand J.D. (2019). Snake Venoms in Drug Discovery: Valuable Therapeutic Tools for Life Saving. Toxins.

[69] Anandhan Sujatha V., Gopalakrishnan C., Anbarasu A., Ponnusamy C.S., Choudhary R., Saravanan Geetha S.A., Ramalingam R. (2024). Beyond the Venom: Exploring the Antimicrobial Peptides from *Androctonus* Species of Scorpion. J. Pept. Sci..

[70] Wang J., Liu X., Song Y., Liu Z., Tang X., Tan H. (2025). LC-AMP-I1, a Novel Venom-Derived Antimicrobial Peptide from the Wolf Spider. Lycosa Coelestis. Antimicrob. Agents Chemother..

[71] Dutta P., Sahu R.K., Dey T., Lahkar M.D., Manna P., Kalita J. (2019). Beneficial Role of Insect-Derived Bioactive Components Against Inflammation and Its Associated Complications (Colitis and Arthritis) and Cancer. Chem. Biol. Interact..

[72] Bulet P., Urge L., Ohresser S., Hetru C., Otvos L. (1996). Enlarged Scale Chemical Synthesis and Range of Activity of Drosocin, an O-Glycosylated Antibacterial Peptide of *Drosophila*. Eur. J. Biochem..

[73] Liu F.-F., Ding C., Yang L.-L., Li H., Rao X.-J. (2021). Identification and Analysis of Two Lebocins in the Oriental Armyworm *Mythimna separata*. Dev. Comp. Immunol..

[74] Li J., Hu S., Jian W., Xie C., Yang X. (2021). Plant Antimicrobial Peptides: Structures, Functions, and Applications. Bot. Stud..

[75] Tam J., Wang S., Wong K., Tan W. (2015). Antimicrobial Peptides from Plants. Pharmaceuticals.

[76] Darbandi A., Asadi A., Mahdizade Ari M., Ohadi E., Talebi M., Halaj Zadeh M., Darb Emamie A., Ghanavati R., Kakanj M. (2022). Bacteriocins: Properties and Potential Use as Antimicrobials. Clin. Lab. Anal..

[77] Zimina M., Babich O., Prosekov A., Sukhikh S., Ivanova S., Shevchenko M., Noskova S. (2020). Overview of Global Trends in Classification, Methods of Preparation and Application of Bacteriocins. Antibiotics.

[78] Soltani S., Hammami R., Cotter P.D., Rebuffat S., Said L.B., Gaudreau H., Bédard F., Biron E., Drider D., Fliss I. (2021). Bacteriocins as a New Generation of Antimicrobials: Toxicity Aspects and Regulations. FEMS Microbiol. Rev..

[79] Negash A.W., Tsehai B.A. (2020). Current Applications of Bacteriocin. Int. J. Microbiol..

[80] Benítez-Chao D.F., León-Buitimea A., Lerma-Escalera J.A., Morones-Ramírez J.R. (2021). Bacteriocins: An Overview of Antimicrobial, Toxicity, and Biosafety Assessment by in Vivo Models. Front. Microbiol..

[81] Ismael M., Huang M., Zhong Q. (2024). The Bacteriocins Produced by Lactic Acid Bacteria and the Promising Applications in Promoting Gastrointestinal Health. Foods.

[82] Tajbakhsh M., Karimi A., Fallah F., Akhavan M.M. (2017). Overview of Ribosomal and Non-Ribosomal Antimicrobial Peptides Produced by Gram Positive Bacteria. Cell Mol. Biol. (Noisy-Le-Grand).

[83] Duban M., Cociancich S., Leclère V. (2022). Nonribosomal Peptide Synthesis Definitely Working Out of the Rules. Microorganisms.

[84] Zhang L., Wang C., Chen K., Zhong W., Xu Y., Molnár I. (2023). Engineering the Biosynthesis of Fungal Nonribosomal Peptides. Nat. Prod. Rep..

[85] Bann S.J., Ballantine R.D., Cochrane S.A. (2021). The Tridecaptins: Non-Ribosomal Peptides That Selectively Target Gram-Negative Bacteria. RSC Med. Chem..

[86] Abdel Monaim S.A., Somboro A.M., El-Faham A., de la Torre B.G., Albericio F. (2019). Bacteria Hunt Bacteria Through an Intriguing Cyclic Peptide. ChemMedChem.

[87] Nuske M.R., Zhong J., Huang R., Sarojini V., Chen J.L.Y., Squire C.J., Blaskovich M.A.T., Leung I.K.H. (2025). Adjuvant Strategies to Tackle *Mcr*-Mediated Polymyxin Resistance. RSC Med. Chem..

[88] Slingerland C.J., Martin N.I. (2024). Recent Advances in the Development of Polymyxin Antibiotics: 2010–2023. ACS Infect. Dis..

[89] Machushynets N.V., Al Ayed K., Terlouw B.R., Du C., Buijs N.P., Willemse J., Elsayed S.S., Schill J., Trebosc V., Pieren M. (2024). Discovery and Derivatization of Tridecaptin Antibiotics with Altered Host Specificity and Enhanced Bioactivity. ACS Chem. Biol..

[90] Zhu S. (2008). Discovery of Six Families of Fungal Defensin-Like Peptides Provides Insights into Origin and Evolution of the CSαβ Defensins. Mol. Immunol..

[91] Hou X., Sun R., Feng Y., Zhang R., Zhu T., Che Q., Zhang G., Li D. (2022). Peptaibols: Diversity, Bioactivity, and Biosynthesis. Eng. Microbiol..

[92] Gavryushina I.A., Georgieva M.L., Kuvarina A.E., Sadykova V.S. (2021). Peptaibols as Potential Antifungal and Anticancer Antibiotics: Current and Foreseeable Development (Review). Appl. Biochem. Microbiol..

[93] Víglaš J., Dobiasová S., Viktorová J., Ruml T., Repiská V., Olejníková P., Gbelcová H. (2021). Peptaibol-Containing Extracts of *Trichoderma atroviride* and the Fight Against Resistant Microorganisms and Cancer Cells. Molecules.

[94] Talapko J., Škrlec I. (2020). The Principles, Mechanisms, and Benefits of Unconventional Agents in the Treatment of Biofilm Infection. Pharmaceuticals.

[95] Kalelkar P.P., Riddick M., García A.J. (2021). Biomaterial-Based Antimicrobial Therapies for the Treatment of Bacterial Infections. Nat. Rev. Mater..

[96] Lima P.G., Oliveira J.T.A., Amaral J.L., Freitas C.D.T., Souza P.F.N. (2021). Synthetic Antimicrobial Peptides: Characteristics, Design, and Potential as Alternative Molecules to Overcome Microbial Resistance. Life Sci..

[97] Sarkar T., Chetia M., Chatterjee S. (2021). Antimicrobial Peptides and Proteins: From Nature’s Reservoir to the Laboratory and Beyond. Front. Chem..

[98] Svenson J., Molchanova N., Schroeder C.I. (2022). Antimicrobial Peptide Mimics for Clinical Use: Does Size Matter?. Front. Immunol..

[99] Lata M., Telang V., Gupta P., Pant G., Kalyan M., Arockiaraj J., Pasupuleti M. (2024). Synthetic Short Cryptic Antimicrobial Peptides as Templates for the Development of Novel Biotherapeutics Against WHO Priority Pathogen. Int. J. Pept. Res. Ther..

[100] Yan J., Cai J., Zhang B., Wang Y., Wong D.F., Siu S.W.I. (2022). Recent Progress in the Discovery and Design of Antimicrobial Peptides Using Traditional Machine Learning and Deep Learning. Antibiotics.

[101] Saubi C., Carratalá J.V., Bello-Madruga R., López-Cano A., Navarro S., Arís A., Garcia-Fruitós E. (2025). Evaluating Host Defense Peptides: A Comparative Analysis of Synthetic Peptides and Recombinant Concatemers. Biomolecules.

[102] Mahlapuu M., Björn C., Ekblom J. (2020). Antimicrobial Peptides as Therapeutic Agents: Opportunities and Challenges. Crit. Rev. Biotechnol..

[103] Han Y., Zhang M., Lai R., Zhang Z. (2021). Chemical Modifications to Increase the Therapeutic Potential of Antimicrobial Peptides. Peptides.

[104] Qi Y.-K., Zheng J.-S., Liu L. (2024). Mirror-Image Protein and Peptide Drug Discovery Through Mirror-Image Phage Display. Chem.

[105] Xu S., Tan P., Tang Q., Wang T., Ding Y., Fu H., Zhang Y., Zhou C., Song M., Tang Q. (2023). Enhancing the Stability of Antimicrobial Peptides: From Design Strategies to Applications. Chem. Eng. J..

[106] Jin K., Sam I., Po K.H.L., Lin D.A., Ghazvini Zadeh E.H., Chen S., Yuan Y., Li X. (2016). Total synthesis of teixobactin. Nat. Commun..

[107] Qi Y.K., Tang X., Wei N.N., Pang C.J., Du S.S., Wang K. (2022). Discovery, synthesis, and optimization of teixobactin, a novel antibiotic without detectable bacterial resistance. J. Pept. Sci..

[108] Wibowo D., Zhao C.-X. (2019). Recent Achievements and Perspectives for Large-Scale Recombinant Production of Antimicrobial Peptides. Appl. Microbiol. Biotechnol..

[109] Shanmugaraj B., Bulaon C.J.I., Malla A., Phoolcharoen W. (2021). Biotechnological Insights on the Expression and Production of Antimicrobial Peptides in Plants. Molecules.

[110] Mazurkiewicz-Pisarek A., Baran J., Ciach T. (2023). Antimicrobial Peptides: Challenging Journey to the Pharmaceutical, Biomedical, and Cosmeceutical Use. Int. J. Mol. Sci..

[111] Zhang Q. (2025). Antimicrobial Peptides: From Discovery to Developmental Applications. Appl. Environ. Microbiol..

[112] Asif F., Zaman S.U., Arnab M.K., Hasan M., Islam M.M. (2024). Antimicrobial Peptides as Therapeutics: Confronting Delivery Challenges to Optimize Efficacy. Microbe.

[113] Arsene M.M., Jorelle A.B., Sarra S., Viktorovna P.I., Davares A.K., Ingrid N.K., Steve A.A., Andreevna S.L., Vyacheslavovna Y.N., Carime B.Z. (2022). Short Review on the Potential Alternatives to Antibiotics in the Era of Antibiotic Resistance. J. Appl. Pharm. Sci..

[114] Divyashree M., Mani M.K., Reddy D., Kumavath R., Ghosh P., Azevedo V., Barh D. (2020). Clinical Applications of Antimicrobial Peptides (AMPs): Where Do We Stand Now?. Protein Pept. Lett..

[115] Al Musaimi O. (2025). FDA-Approved Antibacterials and Echinocandins. Antibiotics.

[116] Baindara P., Kumari S., Dinata R., Mandal S.M. (2025). Antimicrobial Peptides: Evolving Soldiers in the Battle against Drug-Resistant Superbugs. Mol. Biol. Rep..

[117] Duong L., Gross S.P., Siryaporn A. (2021). Developing Antimicrobial Synergy with AMPs. Front. Med. Technol..

[118] Mhlongo J.T., Waddad A.Y., Albericio F., De La Torre B.G. (2023). Antimicrobial Peptide Synergies for Fighting Infectious Diseases. Adv. Sci..

[119] Zharkova M.S., Orlov D.S., Golubeva O.Y., Chakchir O.B., Eliseev I.E., Grinchuk T.M., Shamova O.V. (2019). Application of Antimicrobial Peptides of the Innate Immune System in Combination with Conventional Antibiotics—A Novel Way to Combat Antibiotic Resistance?. Front. Cell. Infect. Microbiol..

[120] Kampshoff F., Willcox M.D.P., Dutta D. (2019). A Pilot Study of the Synergy between Two Antimicrobial Peptides and Two Common Antibiotics. Antibiotics.

[121] Taheri-Araghi S. (2024). Synergistic Action of Antimicrobial Peptides and Antibiotics: Current Understanding and Future Directions. Front. Microbiol..

[122] Xiao G., Li J., Sun Z. (2023). The Combination of Antibiotic and Non-Antibiotic Compounds Improves Antibiotic Efficacy Against Multidrug-Resistant Bacteria. Int. J. Mol. Sci..

[123] Dhanda G., Acharya Y., Haldar J. (2023). Antibiotic Adjuvants: A Versatile Approach to Combat Antibiotic Resistance. ACS Omega.

[124] Ye Z., Fu L., Li S., Chen Z., Ouyang J., Shang X., Liu Y., Gao L., Wang Y. (2024). Synergistic Collaboration between AMPs and Non-Direct Antimicrobial Cationic Peptides. Nat. Commun..

[125] Li J., Fernández-Millán P., Boix E. (2020). Synergism between Host Defence Peptides and Antibiotics Against Bacterial Infections. Curr. Top. Med. Chem..

[126] Shemyakin I.G., Firstova V.V., Fursova N.K., Abaev I.V., Filippovich S.Y., Ignatov S.G., Dyatlov I.A. (2020). Next-Generation Antibiotics, Bacteriophage Endolysins, and Nanomaterials for Combating Pathogens. Biochem. Mosc..

[127] Hemmati S., Saeidikia Z., Seradj H., Mohagheghzadeh A. (2024). Immunomodulatory Peptides as Vaccine Adjuvants and Antimicrobial Agents. Pharmaceuticals.

[128] Panjla A., Kaul G., Chopra S., Titz A., Verma S. (2021). Short Peptides and Their Mimetics as Potent Antibacterial Agents and Antibiotic Adjuvants. ACS Chem. Biol..

[129] Xing H., Loya-Perez V., Franzen J., Denton P.W., Conda-Sheridan M., Rodrigues De Almeida N. (2023). Designing Peptide Amphiphiles as Novel Antibacterials and Antibiotic Adjuvants Against Gram-Negative Bacteria. Bioorganic Med. Chem..

[130] Lu T., Zheng X., Mao F., Cao Q., Cao Q., Zhu J., Li X., Lan L., Li B., Li J. (2022). Novel Niclosamide-Derived Adjuvants Elevating the Efficacy of Polymyxin B Against MDR *Pseudomonas Aeruginosa* DK2. Eur. J. Med. Chem..

[131] Lu T., Han H., Wu C., Li Q., Hu H., Liu W., Shi D., Chen F., Lan L., Li J. (2025). Discovery of a Novel Polymyxin Adjuvant Against Multidrug-Resistant Gram-Negative Bacteria Through Oxidative Stress Modulation. Acta Pharm. Sin. B.

[132] Taylor S.D., Palmer M. (2016). The Action Mechanism of Daptomycin. Bioorg. Med. Chem..

[133] Lev K., Kunz Coyne A.J., Kebriaei R., Morrisette T., Stamper K., Holger D.J., Canfield G.S., Duerkop B.A., Arias C.A., Rybak M.J. (2022). Evaluation of Bacteriophage-Antibiotic Combination Therapy for Biofilm-Embedded MDR *Enterococcus faecium*. Antibiotics.

[134] Silva A.R.P., Guimarães M.S., Rabelo J., Belén L.H., Perecin C.J., Farías J.G., Santos J.H.P.M., Rangel-Yagui C.O. (2022). Recent advances in the design of antimicrobial peptide conjugates. J. Mater. Chem. B..

[135] Sun H., Hong Y., Xi Y., Zou Y., Gao J., Du J. (2018). Synthesis, Self-Assembly, and Biomedical Applications of Antimicrobial Peptide–Polymer Conjugates. Biomacromolecules.

[136] Singh A.K., Kumar A., Singh H., Sonawane P., Paliwal H., Thareja S., Pathak P., Grishina M., Jaremko M., Emwas A.-H. (2022). Concept of Hybrid Drugs and Recent Advancements in Anticancer Hybrids. Pharmaceuticals.

[137] Alkhzem A.H., Woodman T.J., Blagbrough I.S. (2022). Design and Synthesis of Hybrid Compounds as Novel Drugs and Medicines. RSC Adv..

[138] Lee J., Seo C.H., Luchian T., Park Y. (2016). Antimicrobial Peptide CMA3 Derived from the CA-MA Hybrid Peptide: Antibacterial and Anti-Inflammatory Activities with Low Cytotoxicity and Mechanism of Action in Escherichia Coli. Antimicrob. Agents Chemother..

[139] Kim H., Jang J.H., Kim S.C., Cho J.H. (2020). Development of a Novel Hybrid Antimicrobial Peptide for Targeted Killing of *Pseudomonas aeruginosa*. Eur. J. Med. Chem..

[140] Kim H., Jang J.H., Kim S.C., Cho J.H. (2016). Enhancement of the Antimicrobial Activity and Selectivity of GNU7 Against Gram-Negative Bacteria by Fusion with LPS-Targeting Peptide. Peptides.

[141] Cui Z., Luo Q., Bannon M.S., Gray V.P., Bloom T.G., Clore M.F., Hughes M.A., Crawford M.A., Letteri R.A. (2021). Molecular Engineering of Antimicrobial Peptide (AMP)–Polymer Conjugates. Biomater. Sci..

[142] Li C., Li T., Tian X., An W., Wang Z., Han B., Tao H., Wang J., Wang X. (2024). Research Progress on the PEGylation of Therapeutic Proteins and Peptides (TPPs). Front. Pharmacol..

[143] Bellotto O., Semeraro S., Bandiera A., Tramer F., Pavan N., Marchesan S. (2022). Polymer Conjugates of Antimicrobial Peptides (AMPs) with d-Amino Acids (d-Aa): State of the Art and Future Opportunities. Pharmaceutics.

[144] Patrulea V., Gan B.-H., Perron K., Cai X., Abdel-Sayed P., Sublet E., Ducret V., Nerhot N.P., Applegate L.A., Borchard G. (2022). Synergistic Effects of Antimicrobial Peptide Dendrimer-Chitosan Polymer Conjugates Against *Pseudomonas aeruginosa*. Carbohydr. Polym..

[145] Zhang L., Bulaj G. (2012). Converting Peptides into Drug Leads by Lipidation. Curr. Med. Chem..

[146] Bellavita R., Braccia S., Galdiero S., Falanga A. (2023). Glycosylation and Lipidation Strategies: Approaches for Improving Antimicrobial Peptide Efficacy. Pharmaceuticals.

[147] Rounds T., Straus S.K. (2020). Lipidation of Antimicrobial Peptides as a Design Strategy for Future Alternatives to Antibiotics. Int. J. Mol. Sci..

[148] Myšková A., Sýkora D., Kuneš J., Maletínská L. (2023). Lipidization as a Tool Toward Peptide Therapeutics. Drug Deliv..

[149] Makowska M., Kosikowska-Adamus P., Zdrowowicz M., Wyrzykowski D., Prahl A., Sikorska E. (2023). Lipidation of Naturally Occurring α-Helical Antimicrobial Peptides as a Promising Strategy for Drug Design. Int. J. Mol. Sci..

[150] Tortorella A., Leone L., Lombardi A., Pizzo E., Bosso A., Winter R., Petraccone L., Del Vecchio P., Oliva R. (2023). The Impact of N-Glycosylation on the Properties of the Antimicrobial Peptide LL-III. Sci. Rep..

[151] Yang Z., He S., Wu H., Yin T., Wang L., Shan A. (2021). Nanostructured Antimicrobial Peptides: Crucial Steps of Overcoming the Bottleneck for Clinics. Front. Microbiol..

[152] Carmona-Ribeiro A.M., Araújo P.M. (2021). Antimicrobial Polymer−Based Assemblies: A Review. Int. J. Mol. Sci..

[153] Skwarecki A.S., Milewski S., Schielmann M., Milewska M.J. (2016). Antimicrobial Molecular Nanocarrier–Drug Conjugates. Nanomed. Nanotechnol. Biol. Med..

[154] Copling A., Akantibila M., Kumaresan R., Fleischer G., Cortes D., Tripathi R.S., Carabetta V.J., Vega S.L. (2023). Recent Advances in Antimicrobial Peptide Hydrogels. Int. J. Mol. Sci..

[155] Huang Y., Zou L., Wang J., Jin Q., Ji J. (2022). Stimuli-responsive Nanoplatforms for Antibacterial Applications. WIREs Nanomed. Nanobiotechnol..

[156] Guerrero M., Filho D., Ayala A.N., Rafael D., Andrade F., Marican A., Vijayakumar S., Durán-Lara E.F. (2025). Hydrogel-Antimicrobial Peptide Association: A Novel and Promising Strategy to Combat Resistant Infections. Colloids Surf. B Biointerfaces.

[157] Li Y., Han Y., Li H., Niu X., Zhang D., Wang K. (2024). Antimicrobial Hydrogels: Potential Materials for Medical Application. Small.

[158] Borro B.C., Nordström R., Malmsten M. (2020). Microgels and Hydrogels as Delivery Systems for Antimicrobial Peptides. Colloids Surf. B Biointerfaces.

[159] Pulat G., Çelebi N.N., Bilgiç E. (2025). The Effect of Immobilization Methods of P9-4 Antimicrobial Peptide onto Gelatin Methacrylate on Multidrug-Resistant Bacteria: A Comparative Study. Macromol. Biosci..

[160] Sharmin S., Rahaman M.M., Sarkar C., Atolani O., Islam M.T., Adeyemi O.S. (2021). Nanoparticles as Antimicrobial and Antiviral Agents: A Literature-Based Perspective Study. Heliyon.

[161] Gakiya-Teruya M., Palomino-Marcelo L., Pierce S., Angeles-Boza A.M., Krishna V., Rodriguez-Reyes J.C.F. (2020). Enhanced Antimicrobial Activity of Silver Nanoparticles Conjugated with Synthetic Peptide by Click Chemistry. J. Nanopart. Res..

[162] Zharkova M.S., Golubeva O.Y., Orlov D.S., Vladimirova E.V., Dmitriev A.V., Tossi A., Shamova O.V. (2021). Silver Nanoparticles Functionalized with Antimicrobial Polypeptides: Benefits and Possible Pitfalls of a Novel Anti-Infective Tool. Front. Microbiol..

[163] Zheng K., Setyawati M.I., Lim T.-P., Leong D.T., Xie J. (2016). Antimicrobial Cluster Bombs: Silver Nanoclusters Packed with Daptomycin. ACS Nano.

[164] Gao J., Na H., Zhong R., Yuan M., Guo J., Zhao L., Wang Y., Wang L., Zhang F. (2020). One Step Synthesis of Antimicrobial Peptide Protected Silver Nanoparticles: The Core-Shell Mutual Enhancement of Antibacterial Activity. Colloids Surf. B Biointerfaces.

[165] Xu J., Li Y., Wang H., Zhu M., Feng W., Liang G. (2021). Enhanced Antibacterial and Anti-Biofilm Activities of Antimicrobial Peptides Modified Silver Nanoparticles. Int. J. Nanomed..

[166] Boge L., Hallstensson K., Ringstad L., Johansson J., Andersson T., Davoudi M., Larsson P.T., Mahlapuu M., Håkansson J., Andersson M. (2019). Cubosomes for topical delivery of the antimicrobial peptide LL-37. Eur. J. Pharm. Biopharm..

[167] Yi R., Shi Y., Cao X., Pan C. (2025). Actinomycetes: Treasure Trove for Discovering Novel Antibiotic Candidates. Eur. J. Med. Chem..

