# Supplementary material for: Current Status of the Application of Antimicrobial Peptides and Their Conjugated Derivatives
Source: Molecules. 2025 Jul 22;30(15):3070. doi: 10.3390/molecules30153070 (PMC12348590; doi:10.3390/molecules30153070)
Supplement: Supplementary file 1 [file molecules-30-03070-s001.zip › molecules-3771126-supplementary.pdf]

Current Status of the Application of Antimicrobial Peptides and their Conjugated Derivatives

Marcel·lí del Olmo [1] and Cecilia Andreu[2,*]

[1] Departament de Bioquímica i Biologia Molecular; Universitat de València (UVEG); Doctor Moliner 50; 46100; Burjassot; València; Spain; m.del.olmo@uv.es
[2] Departament de Química Orgànica; Universitat de València (UVEG); Vicent Andrés Estellés sn; 46100; València; Spain; cecilia.andreu@uv.es
* Correspondence: cecilia.andreu@uv.es

Table S1: Antimicrobial peptides of animal origin.

Table S2: Bacterial ribosomal antimicrobial peptides.

Table S3: Microbial Nonribosomal Antimicrobial Peptides.

Table S1: Antimicrobial peptides of animal origin. Information extracted from Antimicrobial Peptide Database APD [1].

NAME/ORIGIN	CHARACTERISTICS	ACTIVITY
GNCP-1/ *Cavia porcellus*	Alpha defensin<br>Length:31; Net charge:7; Hydrophobic residue%:41% ; Boman Index:3.01;<br>3 disulphide bridges	Anti-Gram+, Gram-, Antiviral, Antifungal
Human defensin 5/ Paneth cells, intestine, urinary tract, *Homo sapiens*	Alpha Defensin<br>Length:32; Net charge:4; Hydrophobic residue%:40% ; Boman Index:2.6;<br>3 disulfide bridges.	Anti-Gram+ , Gram-, Antiviral, Antifungal, Anti-toxin, anti-sepsis, Wound healing
eNAP-2/ Horse, *Equus caballus*	Alpha defensin<br>Length:46; Net charge:4; Hydrophobic residue%:28% ; Boman Index:1.98;<br>3 disulfide bonds	Anti-Gram+, Gram-
HaNP-2 / Golden hamster or Syrian hamster, *Mesocricetus auratus*	Alpha-defensin<br>Length:31; Net charge:3; Hydrophobic residue%:38% ; Boman Index:2.32<br>3 disulfide bonds: 1,29; 3,18; 8,28	Anti-Gram+, Gram-, candidacidal
Cryptdin-1 /small intestine, mice, *Mus musculus*	Alpha defensins<br>Length:35; Net charge:9; Hydrophobic residue%:37% ; Boman Index:3.53;<br>3 Disulfid bridges: 6, 34; 8, 23; 13, 33.	Anti-Gram+, Gram-
Bovine Beta-defensin 3/ cattle, Bos taurus	Beta defensin<br>Length:42; Net charge:10; Hydrophobic residue%:38% ; Boman Index:2.68;<br>potentially forming 3 disulfide bonds.	Anti-Gram+, Gram-
Rabbit kidney defensin 1/ kidney, European rabbit, *Oryctolagus cuniculus*	Beta defensin<br>Length:32; Net charge:1; Hydrophobic residue%:40% ; Boman Index:1.53;<br>3 disulfide bridges:3,29; 5,19; 9,28.	Anti-Gram+, Gram-

hBD-26 (human beta-defensin 26)/ *Homo sapiens*	Beta defensin<br>Length:42; Net charge:4; Hydrophobic residue%:42% ; Boman Index:1.58;<br>3 disulfide bridges.	Anti-Gram-
Bovine tracheal antimicrobial peptide/ mucosal epithelial cells, airway, *Bos taurus*	Beta defensin<br>Length:38; Net charge:9; Hydrophobic residue%:42% ; Boman Index:1.71;<br>3 disulfide bridges: 5,34; 12,27; 17,35	Anti-Gram+, Gram-, Antifungal, candidacidal
Chicken Heterophil/ *Gallus gallus*	Beta defensin<br>Length:33; Net charge:4; Hydrophobic residue%:48% ; Boman Index:0.81;<br>3 disulfide bonds	Anti-Gram+, Gram-, Antifungal, candidacidal
Chicken AvBD2/ intestinal epithelium and granulocytes, bone marrow, *Gallus gallus*	Beta defensin<br>Length:36; Net charge:6; Hydrophobic residue%:47% ; Boman Index:0.29;<br>3 disulfide bonds (C3-C29, C8-C23, and C13-C30	Anti-Gram+, Gram-
hBD-26/ *Homo sapiens*	Beta defensin<br>Length:42; Net charge:4; Hydrophobic residue%:42% ; Boman Index:1.58;	Anti-Gram-
BTD-2/ *Papio anubis*	Rigid beta-hairpin structure Length:18; Net charge:6; Hydrophobic residue%:56% ; Boman Index:3.54;<br>3 disulfide bridges:3,29; 5,19; 9,28.	Anti-Gram+, Gram-, Antifungal, candidacidal
BTD-4/ *Papio anubis*	Theta-defensin<br>Length:18; Net charge:3; Hydrophobic residue%:66% ; Boman Index:0.61<br>3 disulfide bonds: 1,29; 3,18; 8,28	Anti-Gram+ & Gram-, Antifungal, candidacidal
Heliomicin/ tobacco budworm, *Heliothis virescens*	3D structure: Combine Helix and Beta; Length:44; Net charge:2; Hydrophobic residue%:36% ; Boman Index:1.74;<br>3 Disulphide bridges:7,32; 18,40; 22,42.	Against C. albicans and P. pastoris.
Bactenecin/ Bovine neutrophils, cattle, *Bos taurus*	Arg-rich, Cyclic, S-S bond, amidation; 3D Structure: Unknown<br>Length:12; Net charge:4; Hydrophobic residue%:66% ; Boman Index:2.52;	Anti-Gram+, Gram-, Synergistic AMPs, Wound healing
Tritrpticin/pig	3D Structure Nonhelixbeta, cathelicidin,<br>Length:13 Net charge:4; Hydrophobic residue%:53% ; Boman Index:2.9;	Anti-Gram+, Gram-, Antifungal, Anti-MRSA, Hemolytic, Anticancer
Protegrin 1/ leukocytes; porcine neutrophil, pig, *Sus scrofa*	Arg-rich; homodimer; 3D Structure: Beta;<br>Length:18 Net charge:7; Hydrophobic residue%:44% ; Boman Index:3.65;<br>Two disulfide bridges: 6,15; 8,13	Anti-Gram+, Antiviral, Antifungal, candidacidal, Anti-HIV, Anti-MRSA, anti-sepsis, Synergistic AMPs, Hemolytic, Antibiofilm
ChBac5 /<br>Leukocytes, Goat *Capra hircus*	Pro-rich; Arg-rich; 3D Structure: Rich<br>Sequence analysis: almost exclusively of X-P-P-X repeats;<br>Length:43 Net charge:10; Hydrophobic residue%:25% ; Boman Index:2.68;	Anti-Gram+, Gram-, anti-sepsis
[1]LL-37/ lymphocytes, Mesenchymal Stem Cells; islets; sweat/skin; airway/lung, saliva; colonic mucosa; bone marrow and testis, *Homo sapiens*; Also *Pan troglodytes*	3D Structure: Helix<br>Length:37 Net charge:6; Hydrophobic residue%:35% ; Boman Index:2.99;<br><br>**Clinical trials**: LL-37 cream enhanced the healing rate of diabetic foot ulcer (DFU) with mild infection, but did not decrease the levels of IL-1 alpha and TNF-alpha and the number of aerobic bacteria colonization. This trial is registered at ClinicalTrials.gov, number NCT04098562. In addition, OP-145, a derivative of LL-37, has been subjected to clinical trials (see AP00945).	Anti-Gram+, Gram-, Antiviral, Antifungal, candidacidal, Antiparasitic, Spermicidal, Anti-HIV, Chemotactic, Anti-MRSA, Enzyme inhibitor, anti-TB, anti-sepsis, Synergistic AMPs, Hemolytic, Antibiofilm, Wound healing, Anticancer

Indolicidin/ bovine neutrophils, cattle, *Bos taurus*	Trp-rich; C-terminal amide; 3D Structure: nonhelixbeta; Length:13 Net charge:4; Hydrophobic residue%:53% ; Boman Index:1.06;	Anti-Gram+, Gram-, Antiviral, Antifungal, Anti-HIV, Anti-MRSA, Hemolytic, Antibiofilm, Wound healing, Anticancer
RL-37/ Rhesus monkey, *Macaca mulatta*	3D Structure: Helix<br>Length:37 Net charge:8; Hydrophobic residue%:35% ; Boman Index:1.78;	Anti-Gram+, Gram-
OaBac5 / Sheep neutrophil extract, *Ovis aries*	Pro-rich; Arg-rich<br>Length:43 Net charge:11; Hydrophobic residue%:25% ; Boman Index:2.82;	Anti-Gram+, Gram-, anti-sepsis
Organgutan ppyLL-37/ Great Ape	3D Structure: Helix<br>Length:37 Net charge:4; Hydrophobic residue%:37% ; Boman Index:3.14	Anti-Gram+, Gram-
NA-CATH/ Chinese cobra, *Naja atra*	Lys-rich; 3D Structure: Helix: Length:34 Net charge:15; Hydrophobic residue%:38% ; Boman Index:2.03	Anti-Gram+, Gram-, Antibiofilm
Cathelicidin-PY/ Skin secretions; frog, *Paa yunnanensis*,	3D Structure: Random coiled in water , became helical in membrane-mimetic<br>Length:29; Net charge:7; Hydrophobic residue%:37% ; Boman Index:2.39	Anti-Gram+, Gram-, Antifungal, candidacidal, Anti-inflammatory, anti-sepsis
Macaque histatin/Crab eating macaque, Cynomolgus Monkey, Philippine Monkey and the Long-tailed Macaque, *Macaca fascicularis*	His-rich; 3D Structure: Rich;<br>Length:38; Net charge:13; Hydrophobic residue%:5% ; Boman Index:4.94	Antifungal, candidacidal
human Histatin 5/ salivary glands, *Homo sapiens*	His-rich;3D Structure: Helix<br>Length:24; Net charge:12; Hydrophobic residue%:8% ; Boman Index:4.81	Anti-Gram+ & Gram-, Antiviral, Antifungal, candidacidal, Anti-HIV, Anti-MRSA, Enzyme inhibitor
human Histatin 8/ *Homo sapiens*	His-rich; 3D Structure: Rich<br>Length:12; Net charge:6; Hydrophobic residue%:8% ; Boman Index:4.25	Anti-Gram+ & Gram-, Antifungal, candidacidal
human Histatin 1 / human saliva, *Homo sapiens*	His-rich; 3D Structure: Rich<br>Length:38; Net charge:8; Hydrophobic residue%:10% ; Boman Index:4.29	Antifungal, candidacidal, Chemotactic, Wound healing

[1]Clinical trial

Table S2: Bacterial ribosomal antimicrobial peptides. Information extracted from Antimicrobial Peptide Database APD [1].

NAME/ORIGIN	CHARACTERISTICS	ACTIVITY
[1]Nisin A/ *Streptococcus lactis* (*Lactococcus lactis)*	Two chains; 3D Structure: nonhelixbeta; class I; Chemical modification: amino acid sequence derived from nucleotide and the actual active form is a translationally modified peptide. There are two di-hydroalanines (Dha): S5 and S33; one di-dehydrobutyrine (Dhb): T2; one lanthionine: S3-C7; and four methyllanthionines: T8-C11, T13-C19, T23-C26, and T25-C28; Length: 34; Net charge: 5; Hidrophobic residue%: 44%; Boman Index:0.37.<br>AMPs in use:food preservation: Nisin is a food preservative in use in more than 80 countries (approved by the European Union in 1983 and FDA in 1988)	Anti-Gram+, Antiviral, Spermicidal, Anti-MRSA, Synergistic AMPs, Antibiofilm, Wound healing, Anticancer
Epidermin	3D Structure: unknown; class I a; Chemical modification: Thionines between residues 3-7,16-21,	Anti-Gram+

	beta-methylthionines between 8-11, and Cys 22 is modified to NHCH=CHS and linked to residue 19. In addition, residue 14 is dehydrated; Length: 22; Net charge: 2; Hidrophobic residue%: 45%; Boman Index:0.3.	
Formicin/*Bacillus paralicheniformis* APC 1576	Two chains; 3D Structure: unknow; class I; 71.4% similarity to bacterial 2-chain Haloduracin; Chemical modification: predicted patterns of sidechains conexión: Chain A, FrcA: disulfide bond between C1-C8, lanthionine bridges between S7-C17, T18-C23, and T20-C27; Chain B: lanthionine bridges between T1-C8, T13-C17, S19-C23, T23-C26). T2, S4, S5, and T10 are dehydrated into Dha or Dhb. Length: 28; Net charge: 2; Hidrophobic residue%: 35%; Boman Index: 2.12;	Anti-Gram+
Cacaoidin / Streptomyces cacaoi CA-170360	Ser-rich 30%; glycopeptide; 3D Structure: unknow; Chemical modification: Thioether bonds between S1 and C5, and T18 and C23. In addition, C23 is dehydrated to form a double bond between Calpha and carbonyl carbons. There are likely multiple D-amiino acids. N-terminally motified, S2, T6 dehydrated, Y8 is O-glycosylated, S10, S12, S14, and S16 became D-alanines; Length: 23; Net charge: 0; Hidrophobic residue%: 47%; Boman Index: 0.01	Anti-Gram+, Anti-MRSA
Planosporicin/*Planomonospora alba*	3D Structure: nonhelixbeta; class I a; Chemical modification: Residues T2 and S5 are dehydrated. Lanthionines occur between S3-C7, S13-C20, S18-C23, and S21-C24. One methyllanthionine is proposed between T8 and C11. In addition, the carboxylic acids of E14 and the C-terminus are benzylamidated; Length: 24; Net charge: 2; Hidrophobic residue%: 33%; Boman Index: 0.57	Anti-Gram+; Gram-, Anti-MRSA
Warnerin/ *Staphylococcus warneri* KL-1	3D Structure: unknown; Chemical modification: 80.6% similarity to Epilancin 15X. Structural model proposed three thioether bonds S11-C15, T19-C22, and T21-C24. In addition, S2, TT6, T7, and T27 are dehydrated (XXW4) into Dha for S and Dhb for T; Length: 30; Net charge: 7; Hidrophobic residue%: 40%; Boman Index: 0.84.	Anti-Gram+
Ruminococcin A/ *Ruminococcus gnavus*	3D Structure: unknown; class IIa; the unmodified sequence of this peptide shows 84% similarity to lacticin LMG; Length: 24; Net charge: 1; Hidrophobic residue%: 45%; Boman Index: 0.76.	Anti-Gram+
Lacticin 3147/*Lactococcus lactis* DPC3147	Two-chain; 3D Structure: helix; class I; Chemical modification: D-amino acids; lacticin 3147 A1 has a specific lanthionine bridging pattern which resembles the globular type-B lantibiotic mersacidin, whereas the A2 peptide is a member of the elongated type-A lantibiotic class. In addition, residue L-Ser9 in chain A1 was converted to D-Ala, while both residues L-Ser9 and L-Ser12 are converted to D-Ala residues in chain A2; Length: 30; Net charge: -1; Hidrophobic residue%: 43%; Boman Index: 1.11.	Anti-Gram+, Spermicidal, Synergistic AMPs
Cytolysin/ *Enterococcus faecalis*; human microbiota:gut, symbiont bacteria	3D Structure: unknown; class Ib Length: 21; Net charge: 1; Hidrophobic residue%: 57%; Boman Index: -1.04.	Anti-Gram+, Anti-Gram-, Synergistic AMPs, Hemolytic
Salivaricin A/ *Streptococcus salivarius* 20P3	3D Structure: unknown; class Ib; Chemical modification: one lanthionine and two beta-methyllanthionine residues. Length: 22; Net charge: 1; Hidrophobic residue%: 45%; Boman Index: 1.16.	Anti-Gram+

[1]Pediocin PA-1/*Pediococcus acidilactici* PAC-1.0; also *Lactobacillus plantarum* BM-1; ; lactic acid bacteria	3D Structure: combine helix and beta structure; class IIa; Length: 44; Net charge: 6; Hidrophobic residue%: 34%; Boman Index: 1.12; AMPs in use:food preservation	Anti-Gram+, Anti-Gram-, Spermicidal, Anticancer
Carnobacteriocin B2/ *Carnobacterium piscicola*; lactic acid bacteria	3D Structure: helix; class IIa; Length: 48; Net charge: 4; Hidrophobic residue%: 33%; Boman Index: 1.53.	Anti-Gram+ & Gram-, Synergistic AMPs
Leucocin A/ *Leuconostoc gelidum* UAL-187; *Leuconostoc carnosum* Ta11a; *Leucon ostoc pseudomesenteroides* Q U15; *Leuconostoc pseudomesenteroides* K M432Bz ; lactic acid bacteria	3D Structure: helixbeta; class IIa; Length: 37; Net charge: 4; Hidrophobic residue%: 35%; Boman Index: 1.04.	Anti-Gram+, Anti-Gram-
Mundticin ATO6 /*Enterococcus mundtii* ATO6	3D Structure: unknown; class IIa; Length: 43; Net charge: 4; Hidrophobic residue%: 37%; Boman Index: 0.73.	Anti-Gram+
Lactococcin Z/*Lactococcus lactis* QU7	3D Structure: unknown; class IId; Length: 22; Net charge: 2; Hidrophobic residue%: 45%; Boman Index: 1.14.	Anti-Gram+
Sublancin 168 / *Bacillus subtilis* 168	3D Structure: Helix; class IV; There are two disulfide bonds (C7-C36 and C14-C29). Such bridges confer superior stability to this peptideLength: 37; Net charge: 3; Hidrophobic residue%: 48%; Boman Index: 0.44.	Anti-Gram+
Glycocin F/*Lactiplantibacillus plantarum*;	3D Structure: Helix; class IV; There are two disulfide bonds (C7-C36 and C14-C29). Such bridges confer superior stability to this peptide; Length: 43; Net charge: 4; Hidrophobic residue%: 34%; Boman Index: 0.88<br>**Chemical modification**: Glycosylation at both Ser Cys, modified by an N-acetylglucosamine beta-O-linked to Ser18, and an N-acetylhexosamine S-linked to C-terminal Cys43). Two disulfide bonds: C5-C28 and C12-C21.	Anti-Gram+
UCBB1aA/ *Escherichia coli*, human microbiota:gut, symbiont bacteria	3D Structure: unknown; class 2a microcin; Length: 88; Net charge: 0; Hidrophobic residue%: 39%; Boman Index: 0.56.	Anti-Gram-
Microcin 24 /*Escherichia coli* 2424	3D Structure: unknown; class 2a microcin Length: 74; Net charge: 2; Hidrophobic residue%: 43%; Boman Index: 0.28.	Anti-Gram-
Microcin J25/Escherichia coli	3D Structure: beta; Gly-rich; Cyclic structure lactam; microcins, Length: 21; Net charge: -1; Hidrophobic residue%: 33%; Boman Index: -0.64.	Anti-Gram-
Microcin Capistruin/Burkholderia thailandensis	microcin Length: 19; Net charge: 1; Hidrophobic residue%: 43%; Amphiphilicity Index : 0.32.	Gram+, Gram-
Microcin E492/*Klebsiella pneumoniae* RYC492; human microbiota:gut, symbiont bacteria	3D Structure: unknown; class 2b microcin; siderophore-mocrocin family; Length: 84; Net charge: -4; Hidrophobic residue%: 35%; Boman Index: 0.28.	Anti-Gram-, Anticancer

Microcin B17 */Escherichia coli* , human microbiota:gut, symbiont bacteria	3D Structure: unknown; Gly-rich, class 1 microcins; Length: 43; Net charge: 1; Hidrophobic residue%: 16%; Boman Index: 0.01.	Anti-Gram-
Microcin V (old: Colicin)	3D Structure: unknown; class 2a; Length: 88; Net charge: 0; Hidrophobic residue%: 39%; Boman Index: 0.56.	Anti-Gram-
Microcin 24 (old: colicin 24)/ *Escherichia coli* 2424	3D Structure: unknown; class 2a microcins; Length: 74; Net charge: 2; Hidrophobic residue%: 43%; Boman Index: 0.28.	Anti-Gram-
[2]Citrocin/*Citrobacter pasteurii* and *Citrobacter braakii*	3D Structure: Beta; Length: 19; Net charge: 1; Hidrophobic residue%: 31%; Boman Index: -0.3. Chemical modification: it has an eight aa residue ring formed between the backbone NH of residue Gly1 and the side chain CO of residue Glu8; Feed additive: it is used in animal feed	Anti-Gram-
Pantocin wh-1/*Pantoea dispersa* W18	3D Structure: Unknown; Length: 16; Net charge: 0; Hidrophobic residue%: 68%; Boman Index: -1.52;.	Anti-Gram+, Gram-, anti-TB

[1]Clinical use

[b] Animal feed

Table S3: Microbial Nonribosomal Antimicrobial Peptides. Information extracted from Antimicrobial Peptide Database APD [1].

[1]Gramicidin A /	3D Structure: Helix; Trp-rich; Val-rich; Leu-rich; Length: 15; Net charge: 0; Hidrophobic residue%: 93%; Boman Index: -3.31. Chemical modification:N- and C-termini are chemically modified (CHO at the N-terminus and $NHCH_2CH_2OH$ at the C-terminus). Amino acids 4, 6, 8, 10, and 12 are D-amino acids.<br>Used clinically (topically).	Anti-Gram+, Anti- Gram-, Antiviral, Spermicidal, Anti-HIV, Anticancer
GP-19 /*Xenorhabdus budapestensis* NMC-10, also *Lactobacillus crustorum* MN047	3D Structure: Unknown; Pro-rich; Length: 19; Net charge: 0; Hidrophobic residue%: 31%; Boman Index: -1.	Anti-Gram+, ANTI-Gram-, Antifungal
Fusaricidin A/*Bacillus polymyxa* KT-8	3D Structure: Unknown; lactone; depsipeptide, lipopeptide; Length: 6; Net charge: 0; Hidrophobic residue%: 50%; Boman Index: 0.31. Chemical modification: esterification of carboxylic end; Val2, Asn5, Ala6 are D-amino acids and Thr4 is a D-allo-Thr. N-terminus NH is lipidated.	Anti-Gram+, Antifungal
[1]Daptomycin/ *Streptomyces roseosporus*	3D Structure: nonhelixbeta; lactone; depsipeptide; lipopeptide; Length: 13; Net charge: -3; Hidrophobic residue%: 15%; Boman Index: 3.48; Chemical modification: N-terminus lipidated with. Other nonstandard amino acids include ornithine-6, (2S,3R)-3-methyl-glutamic acid-12, and three D-amino acids at positions N2, A8, and S11.<br>Used clinically Daptomycin was approved by FDA in 2003 to treat soft-tissue infections, and in 2006 to treat S. aureus bacteremia and right-sided endocarditis.	Anti-Gram+
[1]Colistin A=Polymyxin E1;colistin B=Polymyxin E2/ *Paenibacillus polymyxa* var. colistinus	3D Structure: nonhelixbeta; lipopeptide; Length: 10; Net charge: -6; Hidrophobic residue%: 20%; Boman Index: 2.86; Chemical modification: multiple Dab. The molecule is cyclic due to the formation of an amide bond between the amino group of Dab in position 4 and the carboxyl group of the C-terminus. Residue Leu6 is a D-amino acid	Anti-Gram-, anti-sepsis, Synergistic AMPs, Antibiofilm

	and the N-terminus is attached by a fatty acid (Polymyxin E1= colistin A: fatty acid A=6-methyloctanoic acid; Polymyxin E2=colistin B: fatty acid B=6-methylheptanoic acid).<br>Used clinically: Colistin sulfate: for oral and topical use. Sodium colistimethate (CMS): for parenteral and inhalation routes. CMS is a prodrug. Mucosal adjuvants that enhance vaccine-induced antigen-specific immune responses	
[1]Gramicidin S/*Bacillus brevis*	3D Structure: beta; lactone; depsipeptide; lipopeptide; Length: 10; Net charge: 2; Hidrophobic residue%: 60%; Boman Index: -1.27; Chemical modification: The second and seventh residues of this cyclic peptide are Ornithines, an analog of lysine, residue F is a D-amino acid. This is the first cyclic peptide antibiotic used clinically. Gramicidin S was used to treat wounds and is still in use in topical ointments and eye drops.	Anti-Gram+ & Gram-, Antifungal, Spermicidal, Synergistic AMPs, Hemolytic, Antibiofilm
[1]Teixobactin/*Eleftheria terrae*, i-chip technology	3D Structure: beta; Ile-rich, lactone, depsipeptide; Length: 11; Net charge: 2; Hidrophobic residue%: 55%; Boman Index: 0.48; Chemical modification: An ester bond is formed between the backbone C-terminal Ile carboxylic acid and the side chain of Thr8. In addition, the N-terminal phe is methylated. There are 4 D-amino acids and enduracididine.<br>It is a potential lead	Anti-Gram+, Anti-MRSA, anti-TB
Tridecaptin A1/*Paenibacillus terrae*	3D Structure: Nonhelixbeta;lipopeptido; Length: 13; Net charge: 2; Hidrophobic residue%: 45%; Boman Index: 0.7; Chemical modification: Dab amino acid and the N-terminal acyl chain.	Anti-Gram-
[1]Polymyxin B/Bacillus aerosporus Greer	3D Structure: Unknown; lactam, lipopeptides; Length: 10; Net charge: 6; Hidrophobic residue%: 20%; Boman Index: 3.05; Chemical modification: Multiple Dab (2,4-diaminobutanoic acid). The molecule is cyclic due to the formation of an amide bond between the amino group of Dab in position 4 and the carboxyl group of the C-terminus.<br>used clinically: administered parenterally in its active form.	Anti-Gram-, Antifungal, anti-sepsis, Antibiofilm
Brevicidine/ *Brevibacillus laterosporus*	3D Structure: Unknown; lactone; depsipeptide; lipopeptides; Length: 12; Net charge: 4; Hidrophobic residue%: 25%; Boman Index: 1.49; Chemical modification: three ornithines at positions 4,5, and 7.	Anti-Gram-
Clovibactin /*Eleftheria terrae* ssp. carolina	3D Structure: Beta; Leu-rich; depsipeptide, lactone; Length: 8; Net charge: 2; Hidrophobic residue%:63%; Boman Index: -0.49; Chemical modification: Leu2 and Lys3 are D-amino acids, Asn5 is beta-hydroxylated, the hydroxyl group of Asn5 forms an ester bond with the carboxyl group of C-terminal Leu8.	Anti-Gram+, Anti- Gram-, Anti-MRSA
[2]Alamethicin/*Trichoderma viride*	3D Structure: Helix; lactam; peptaibol; fungii; Length: 18; Net charge: -1; Hidrophobic residue%: 66%; Boman Index: -0.68; Chemical modification: alpha-aminoisobutyric acid at positions 2, 4, 7, 9, 12, 15, and 16. The side chain of residue E17 forms an amide bond with proline-1	Anti-Gram+, Antifungal, Antiparasitic, Hemolytic
[2]Tricholongin BI/*Trichoderma longibrachiatum*	3D Structure: Helix; peptaibol; fungii; Length: 12; Net charge: 6; Hidrophobic residue%: 50%; Boman Index: -0.74; Chemical modification:Alpha-aminoisobutyric acid; the N-terminal resididue is acetylated and the C-terminus is a leucinol).	Anti-Gram+, Antifungal

[2]Emericellipsin A/*Emericellopsis alkaline* VKPM F1428	3D Structure: Unknown; peptaibol; fungii; Length: 9; Net charge: 0; Hidrophobic residue%: 56%; Boman Index: -0.7; Chemical modification: P1 is methylated, residue 2 is 2-Amino-4-methyl-6-hydroxy-8-oxo decanoic acid, residue 4 is a methylated Ala (Aib), residue 6 is isovaline, residue 7 is beta-alanine, residue 8 is alaninol, and residue 9 is ethanolamine.	Anti-Gram+, Anti- Gram-, Antifungal, Anticancer

[1]Clinical use
All produced by bacteria except those marked as [2].

References

1. Wang, G.; Li, X.; Wang, Z. APD3: The Antimicrobial Peptide Database as a Tool for Research and Education. *Nucleic Acids Res* **2016**, *44* (D1), D1087–D1093. https://doi.org/10.1093/nar/gkv1278.
